# Synthesis, characterization, *in silico* molecular docking, and antibacterial activities of some new nitrogen-heterocyclic analogues based on a *p*-phenolic unit†

**DOI:** 10.1039/d2ra01794f

**Published:** 2022-04-26

**Authors:** Abdel Haleem M. Hussein, Abu-Bakr A. El-Adasy, Ahmed M. El-Saghier, M. Olish, Aboubakr H. Abdelmonsef

**Affiliations:** Chemistry Department, Faculty of Science, Al-Azhar University Assiut Egypt; Chemistry Department, Faculty of Science, Sohag University 82524 Sohag Egypt; Chemistry Department, Faculty of Science, South Valley University 83523 Qena Egypt aboubakr.ahmed@sci.svu.edu.eg

## Abstract

Nitrogen-containing heterocycles have shown pharmacological properties against various diseases. Herein, in our study, flavoHB enzyme is a highly promising well-validated target for identification of antibacterial inhibitors using *in silico* and *in vitro* techniques. To identify a new class of antimicrobial agents, *N*-(4-hydroxyphenyl)-3-oxobutanamide was utilized as a precursor in the synthesis of several nitrogen-based heterocycles (pyridine, pyrimidine, and pyrazole) attached to *p*-phenolic substrates 2–8. Treatment of 3-oxobutanimide 1 with malononitrile and/or ethyl cyanoacetate in ethanolic piperidine afforded the pyridinone analogues 2a,b. On the other hand, treatment of 1 with arylidene cyanothioacetamide furnished the pyridinthione derivative 3. The reaction of starting material 1 with salicylaldehyde and/or dimethyl formamide dimethyl acetal (DMF-DMA) yielded the pyridinones 4 and 5, respectively. Reaction of 1 with terephthalaldehyde and urea or thiourea gave bis structures 6a,b. The reaction of compound 1 with ethyl isothiocyanate and hydrazine hydrate afforded pyrimidine and pyrazole derivatives 7 and 8, respectively. The structures of newly prepared compounds 2–8 were elucidated using elemental data and spectral analyses such as IR, ^1^H NMR, ^13^C NMR, and MS. In addition, an in-house nitrogen-containing heterocycle analogues library 2–8 was examined and screened *in vitro* for their antibacterial effects against Gram-negative bacteria, *Escherichia coli* and Gram-positive bacteria, *Staphylococcus haemolyticus*, *Kocuria kristinae*, *Enterococcus casseliflavus*, and *Bacillus cereus*. Compounds 6a and 6b have also shown the highest antibacterial activity against all types of bacteria strains tested except *Kocuria kristinae*. Further, the molecular docking study of the newly prepared compounds with the target enzyme flavohemoglobin (flavoHB) was undertaken to explore their potential inhibitory activities. The results of the docking study indicated that compounds 6a and 6b have exerted the highest docking scores against the active site of flavoHB. As a result, the *in vitro* and molecular docking study findings suggested that the compounds 6a and 6b (with pyrimidine moiety, amide linkage, and phenolic substrate) might be potent bacterial flavohemoglobin (flavoHB) inhibitors and they could set a promising starting point for future design of antibacterial agents.

## Abstract 2

Compounds 6a and 6b (with pyrimidine moiety, amide linkage, and phenolic substrate) might be potent bacterial flavohemoglobin (flavoHB) inhibitors and they could set a promising starting point for future design of antibacterial agents.

## Graphical abstract

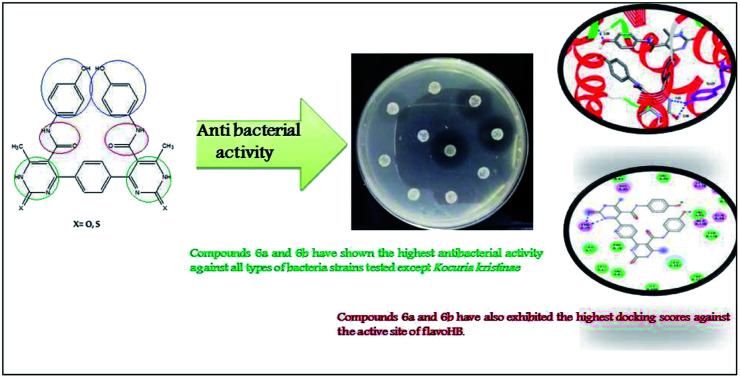

## Introduction

**Fig. 1:**
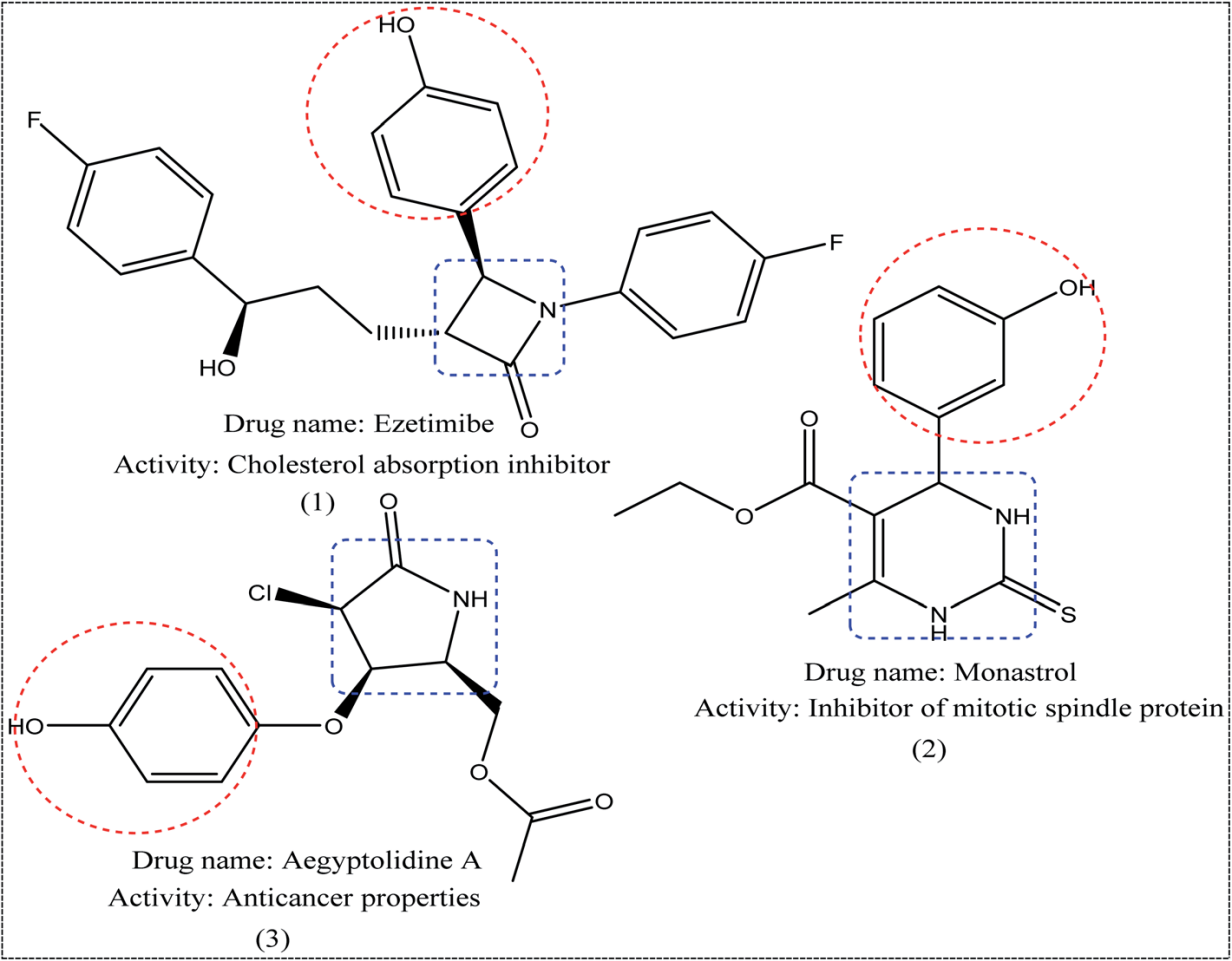
Examples of N-heterocycles-attached to phenolic moiety as clinical drugs.

**Fig. 2:**
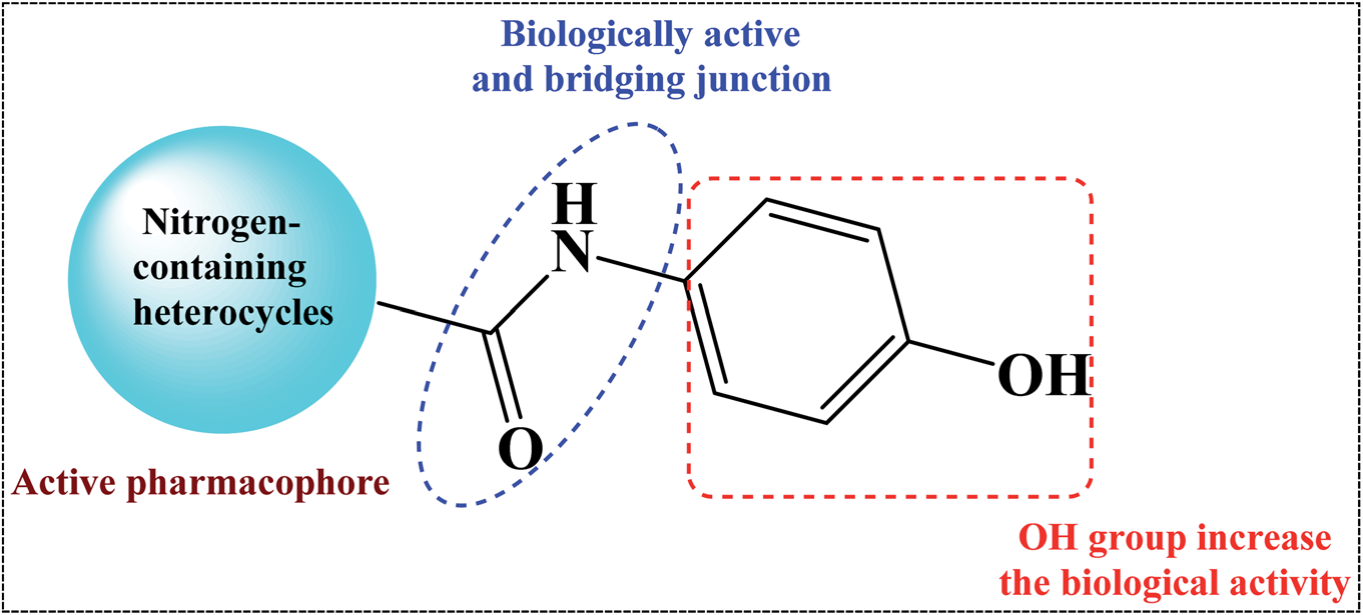
Design of the target compounds.

Nitrogen-containing heterocycles^1–3^ such as pyridine, pyrimidine, and pyrazole are important molecular building blocks that are involved in the structural composition of crucial chemicals for humans, including pharmaceuticals, natural resources, veterinary and agricultural products, analytical reagents, and dyes.^4–6^ In addition, nitrogen-based heterocycles attached to phenolic substrates have received increasing attention due to their pharmacological properties including anticancer, antimalarial, antimicrobial, and antitubercular^6^ activities. The pursuit of these properties requires efficient synthetic routes that allow rapid construction of diverse aromatic heterocycles with defined substitution patterns. Moreover, N-heterocyclic compounds coupled with the phenolic substrate possess a wide range of medicinal and pharmaceutical activities and are constituents of bioactive molecules such as antibiotics, vitamins, and pharmaceutics, as represented in Fig. 1. As aforementioned, there are three pharmacophoric features for good antibacterial activity including an N-heterocyclic system, a linker containing a functional group (amide), and an OH group which forms hydrogen bonds, as shown in Fig. 2.

On the other hand, Helmick *et al.* reported that nitrogen-containing heterocycles such as azoles had antibacterial activity through inhibition of a bacterial heme-containing enzyme called flavohemoglobin (flavoHB).^7^ The flavohemoglobin is a nitric oxide dioxygenase that used for oxidation of nitric oxide to nitrate.^8^ The function of flavohemoglobin is pivotal for the bacterial survival by protecting bacteria from nitrosative stress.^9^ Thus, flavoHB enzyme has been selected as therapeutic target for identification of antibacterial drug candidates.

In our previous work, we have successfully developed the synthesis of hybrid compounds utilizing 3-oxobutanamide derivatives as starting materials.^10–16^ In light of the above information and as part of our ongoing research in synthesis of new hybrid molecules with pharmacological interest,^17–25^ herein, an in-house database of nitrogen-containing heterocycle analogues based *p*-phenolic unit was synthesized, characterized and screened *in vitro* for their antibacterial actions against Gram-negative bacteria, *Escherichia coli* (O157:H7), and Gram-positive bacteria, *Staphylococcus haemolyticus* (050402123720221), *Kocuria kristinae* (050032102000201), *Enterococcus casseliflavus* (120200601563571), and *Bacillus cereus* (MH 656790).

Moreover, the molecular docking studies^26–30^ of the newly prepared compounds with the target enzyme flavohemoglobin (flavoHB) were conducted to explore the potential inhibitory activity of these compounds. Finally, ADMET (absorption, distribution, metabolism, excretion, and toxicity) and drug-likeness predictions of the compounds were calculated using Molinspiration, SwissADME and admetSAR tools. The compounds 6a and 6b (with pyrimidine moiety, amide linkage, and phenolic substrate) showed the strongest antibacterial activities, and exhibited the highest binding affinity to the target enzyme flavohemoglobin (flavoHB) with binding energies Δ*G*s (−10.4 and −10.0 kcal mol^−1^), respectively, higher than of the standard drug.

## Results and discussion

### Chemistry

**Fig. 3:**
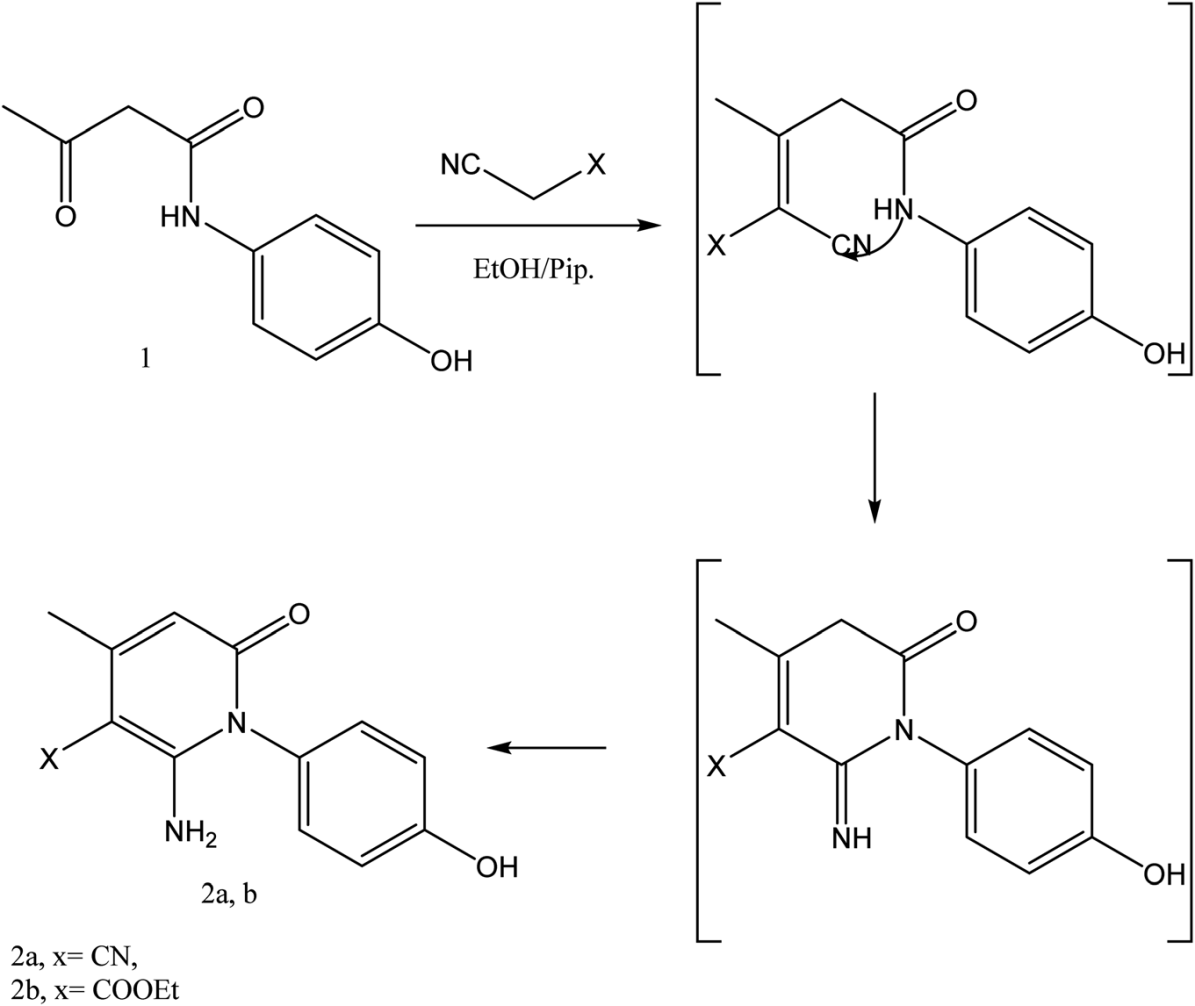
Synthesis of compounds 2a,b.

In the current work, treatment of 3-oxobutanamide 1 with active methylene compounds like malononitrile and ethyl cyanoacetate was achieved as represented in Fig. 3. So, treatment of 1 with malononitrile in ethanolic piperidine solution afforded the pyridinone derivative 2a. IR spectrum of the desired 2a showed disappearing of acetyl carbonyl and appearance one CN at *ν* 2194 cm^−1^. ^1^H NMR of the structure showed singlet signal at *δ* 2.40 ppm assigned to CH_3_, singlet signal for NH_2_ group appeared at *δ* 3.73 ppm, singlet signal corresponding to CH-pyridine at *δ* 7.64 ppm, and OH showed as hump at *δ* 9.33 ppm. Similarly, the reaction of 1 with ethyl cyanoacetate instead of malononitrile, the reaction takes the way itself not on the ester as expected to give compound 2b. The IR spectrum of 2b showed the presence of ester carbonyl band at *ν* 1721 cm^−1^, amide carbonyl at *ν* 1635 cm^−1^ and disappearance of CN absorption band. Moreover, ^1^H-NMR spectrum revealed a triplet and quartet signals at *δ* 1.12 and 4.04 ppm for CH_3_ and CH_2_ groups of esters, respectively. In addition, the other CH_3_ group appeared as a singlet at *δ* 2.28 ppm and appearance of the NH_2_ protons at *δ* 5.14 ppm as a singlet, in addition to CH-pyridine at *δ* 8.12 ppm in interference with the aromatic protons, as well as a singlet signal for OH proton appeared at *δ* 10.32 ppm.

**Fig. 4:**
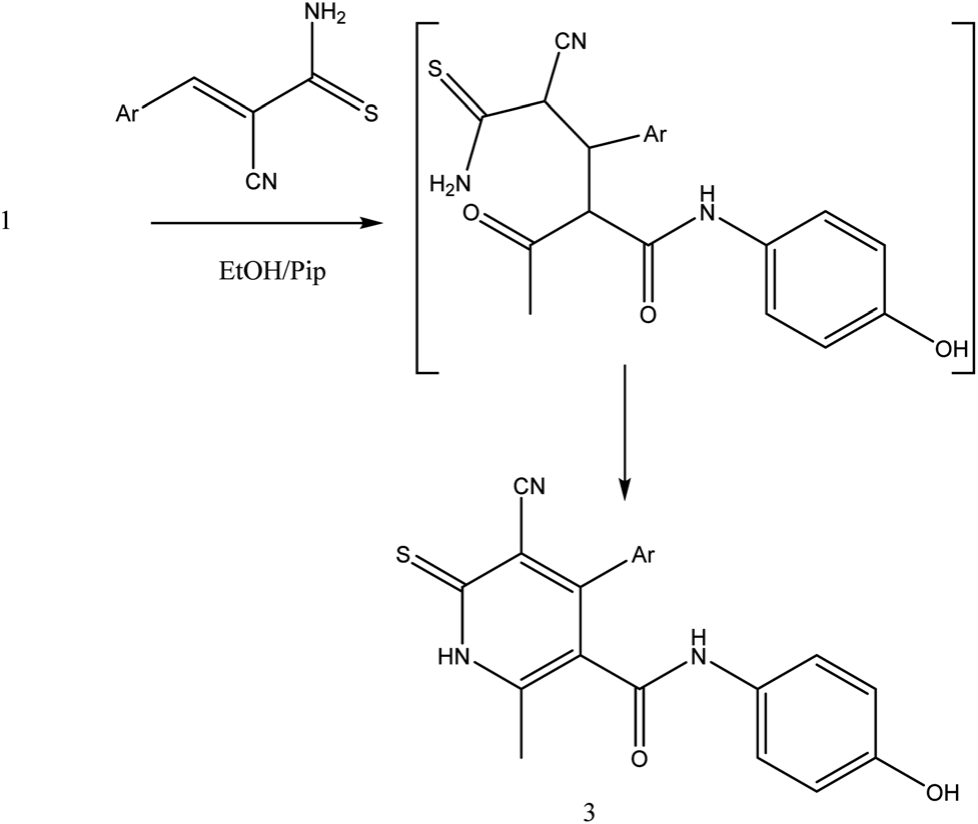
Synthetic pathway of compound 3.

On the other hand, treatment of compound 1 with benzylidene cyanothioacetamide namely, 2-cyano-3-phenylprop-2-enethioamide furnished the pyridinthione derivative 3, as shown in Fig. 4. Its IR spectrum showed band at *ν* 2216 cm^−1^ assigned to CN group, and amide carbonyl at *ν* 1631 cm^−1^. In addition, ^1^H NMR spectrum of compound 3 revealed a singlet signal at *δ* 1.66 ppm assigned to CH_3_, singlet signal at *δ* 3.93 ppm assigned to OH group, NH appeared at *δ* 9.46 ppm and other NH appeared at *δ* 9.83 ppm.

**Fig. 5:**
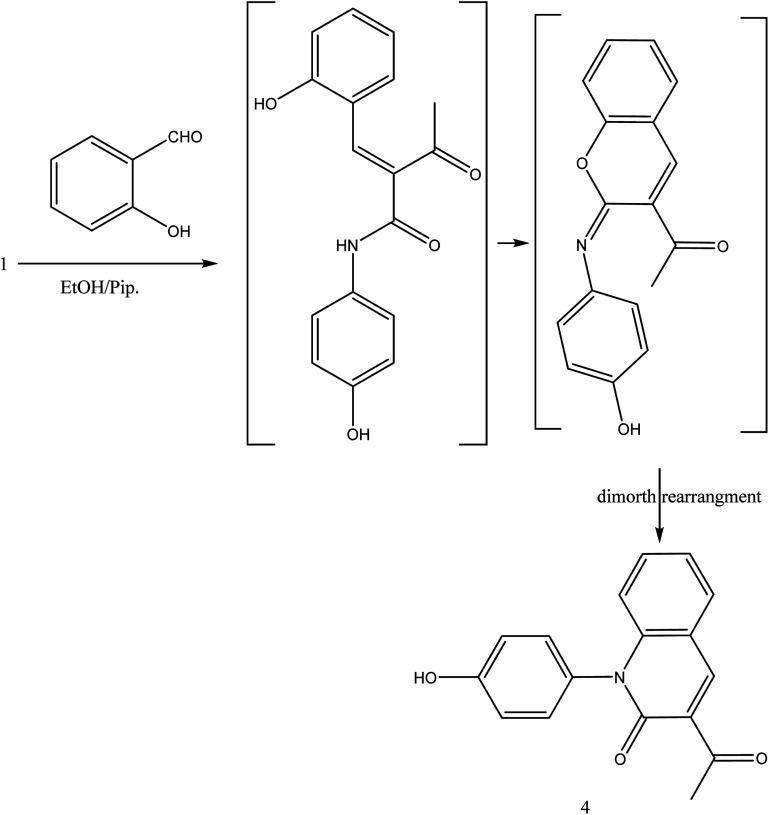
Synthesis of compound 4.

The reaction of 1 with salicylaldehyde takes the same way too, where the formyl group attacks the active methylene of the starting material to furnish the pyridinone 4, as shown in Fig. 5. The IR spectrum showed ester carbonyl at *ν* 1707 cm^−1^ and amide carbonyl at *ν* 1662 cm^−1^. Moreover, ^1^H NMR spectrum showed singlet signal at *δ* 2.38 ppm assigned to CH_3_, *δ* 8.88 ppm CH-pyridine appeared and hydroxyl group appeared at *δ* 9.55 ppm.

**Fig. 6:**
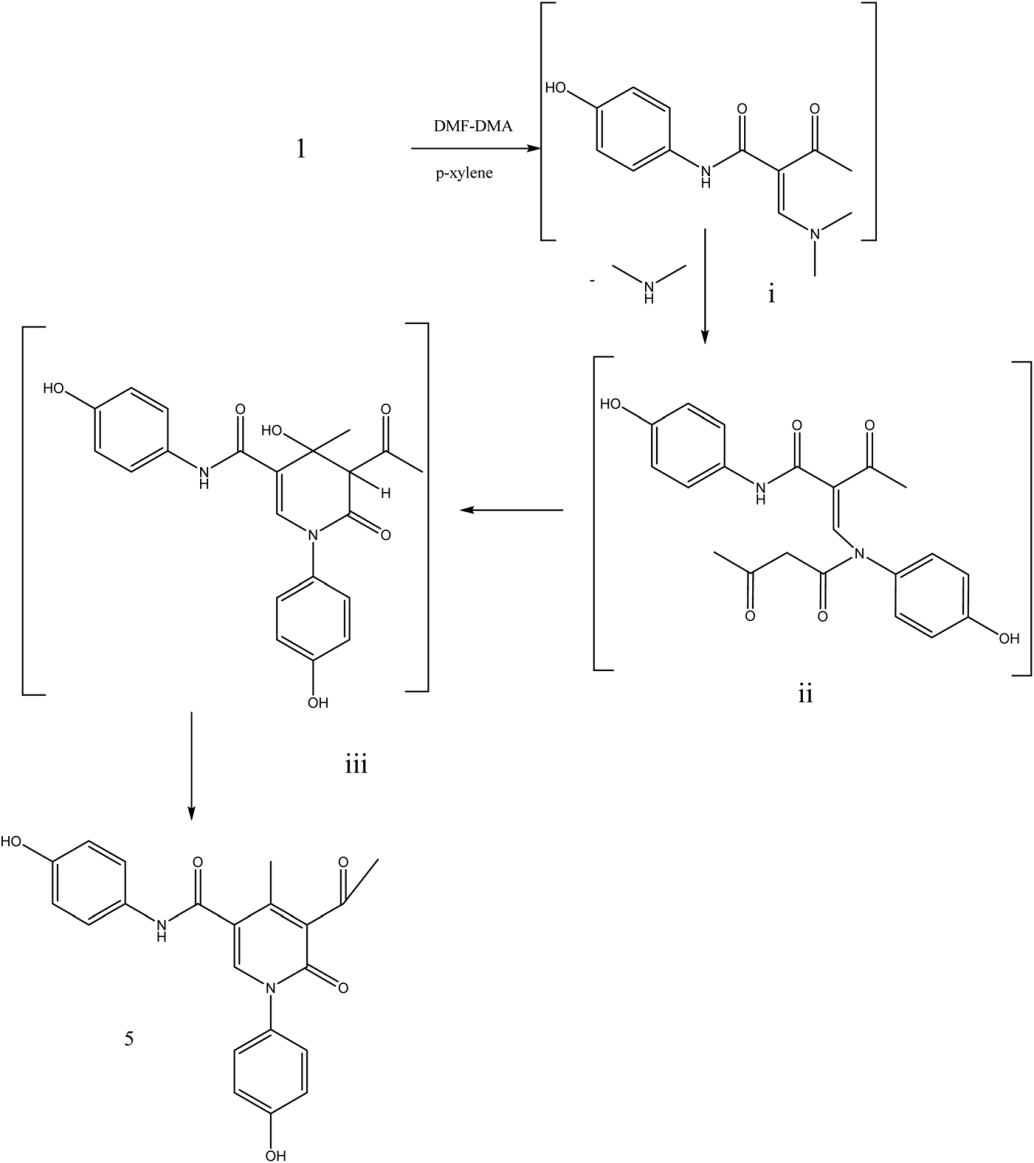
Synthetic strategy of compound 5.

During this phase of our research, we have shown that 3-oxobutanamide 1 is expected to react with dimethylformamide-dimethyl acetal (DMF-DMA) in refluxing dry xylene to yield the product which may be enaminone (i) as literature procedure.^31^ But under the reaction conditions we obtained only a product that could be formulated as 5-acetyl-*N*,1-bis(4-hydroxyphenyl)-4-methyl-6-oxo-1,6-dihydropyridine-3-carboxamide 5. However, the spectral data and chemical evidence did not fit this structure. So, the structure (i) was readily ruled out as final product, but it was formed as intermediate based on spectral data. The mass spectrum showed the molecular ion peak at *m*/*z* = 377 (M^+^) corresponding to molecular formula (C_21_H_18_N_2_O_5_). The IR spectrum of compound 5 showed acetyl carbonyl at *ν* 1710 cm^−1^, and amide carbonyl at *ν* 1650 cm^−1^. ^1^H NMR spectrum revealed singlet signal at *δ* 1.91 ppm referred to acetyl CH_3_, singlet signal at *δ* 2.18 ppm for other CH_3_, CH-pyridine noted at *δ* 8.21 ppm, OH appeared as hump at *δ* 9.50 ppm, and NH appeared at *δ* 11.46 ppm. Moreover, ^13^C NMR spectrum revealed two singlet signals assigned for two CH_3_ at *δ* = 19.73 and 21.47 ppm, three singlet signals assigned for carbonyls at *δ* 164.25, 176.37, 196.46 ppm as declared in Fig. 6.

Formation of compound 5 is assumed to be proceed *via* initial condensation of 3-oxobutanamide 1 with one molecule of DMF-DMA to yield the unstable enaminone (i) which in turn, reacts with another molecule of 1 giving intermediate (ii) by loss NMe_2_NH molecule and cyclized to (iii), then loss H_2_O to yield 5-acetyl-*N*,1-bis(4-hydroxyphenyl)-4-methyl-6-oxo-1,6-dihydropyridine-3-carboxamide 5.

**Fig. 7:**
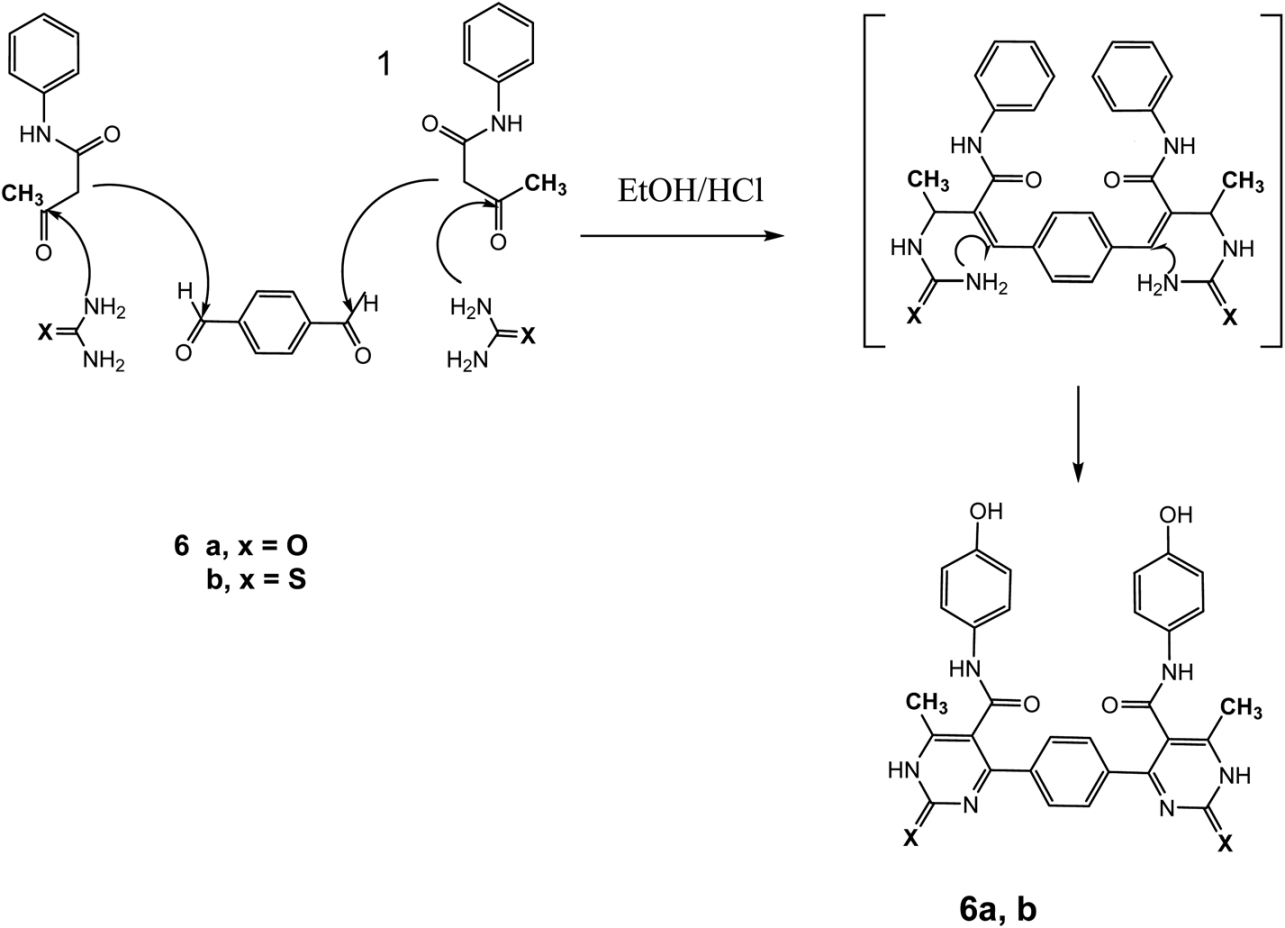
Synthetic pathway of compounds 6a,b.

The reaction of 1 with a mixture of terephthaldehyde and urea or thiourea afforded the bis-structure 6a,b which confirmed on the bases of its compatible spectroscopic data as represented in Fig. 7. IR spectrum of 6a showed two amide carbonyls at *ν* 1662 cm^−1^. ^1^H NMR spectrum showed singlet signal at *δ* 2.28 ppm assigned to CH_3_, singlet signal at *δ* 5.14 ppm assigned to OH group, singlet signal at *δ* 9.61 assigned to NH, and other NH appeared at *δ* 10.32 ppm. ^13^C NMR showed singlet signal at *δ* = 17.62 ppm assigned to CH_3_ group and singlet signal at *δ* = 165.57 ppm assigned to carbonyl group in addition to other carbons in the molecule. Similarly, IR spectrum of compound 6b showed band at *ν* 1686 cm^−1^ for amide carbonyl group. ^1^H NMR spectrum showed singlet signal at *δ* 2.33 ppm assigned to CH_3_ group, singlet signal at *δ* 5.29 ppm for OH group, NH amide appeared at *δ* 8.12 ppm, and NH pyrimidine appeared at *δ* 10.00 ppm. ^13^C NMR declared carbonyl at *δ* = 175 ppm and C=S at 192 ppm, and DEPT 135 revealed disappeared of CH_2_ signal. Mass spectrum showed molecular ion peak at 595 *m*/*z* corresponding to M^−1^ molecular formula C_30_H_24_N_6_O_4_S_2_.

**Fig. 8:**
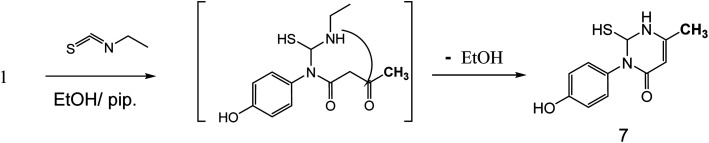
Synthetic route of compound 7.

Also, the reaction of 3-oxobutanamide 1 with ethyl isothiocyanate afforded the pyrimidine derivative 7*via* attaching firstly on amidic NH not on methylene as expected (Fig. 8). Structure 7 was confirmed by compatible spectroscopic data and elemental analysis. ^1^H NMR spectrum of compound 7 showed singlet signal at *δ* = 1.10 ppm assigned to CH_3_, aromatic protons appeared at *δ* 6.89–7.27 ppm as doublet of doublet signals, CH-pyrimidine appeared at 9.32 ppm, and broad signals appeared at *δ* 9.77 and 10.15 ppm referred to SH, and OH groups, respectively. ^13^C NMR confirmed the presences of CH_3_ signal at *δ* = 14.82 ppm, and one carbonyl at *δ* = 165.72 ppm, in addition to other carbons in the structure. Moreover, mass spectrum showed the molecular ion peak at 236 *m*/*z* corresponding to molecular formula C_11_H_12_N_2_O_2_S.

**Fig. 9:**
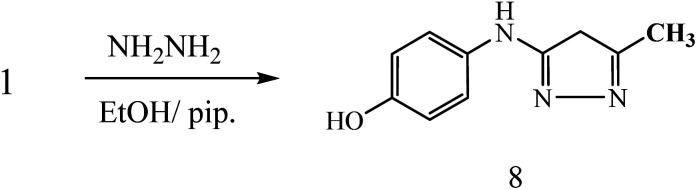
Synthesis of compound 8.

**Fig. 10:**
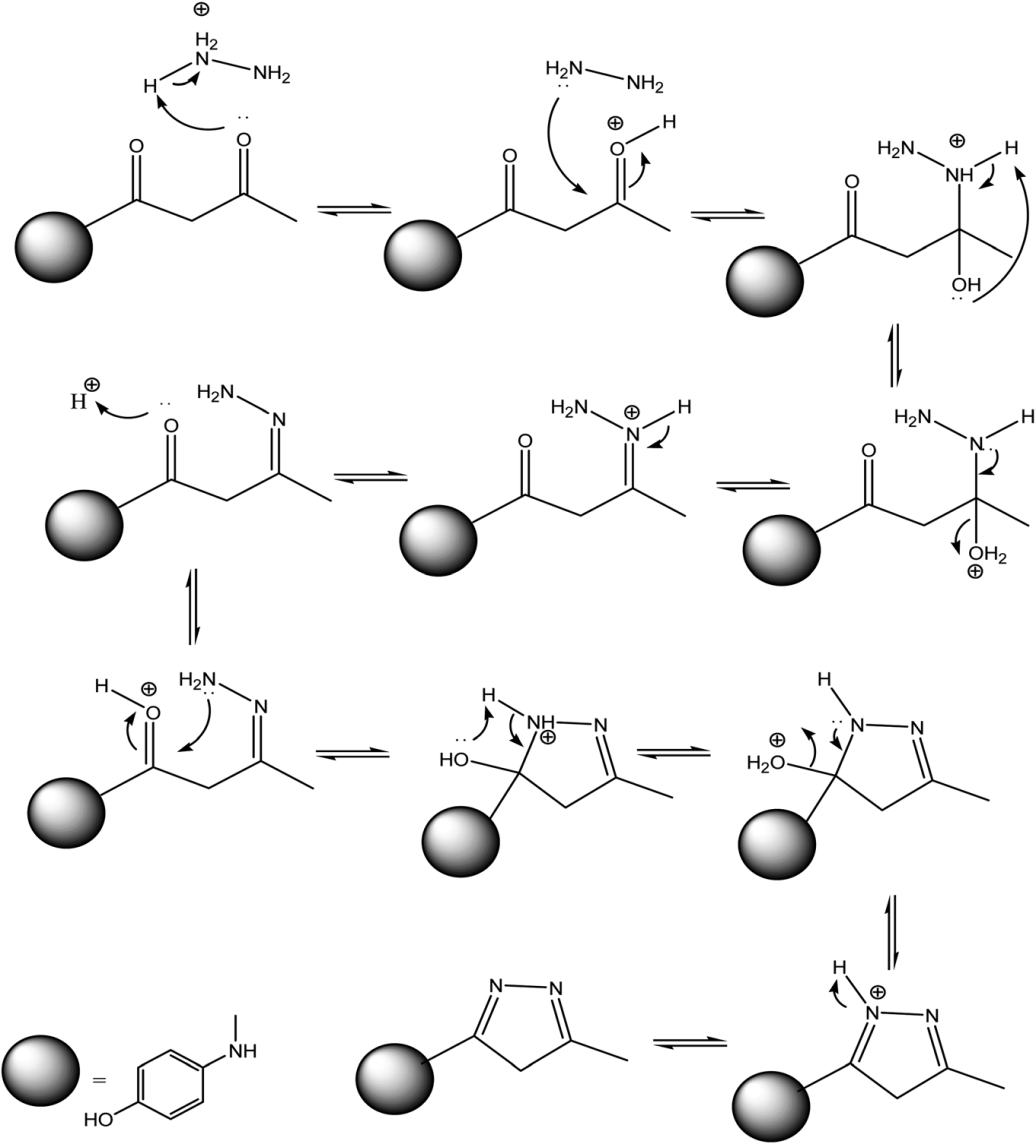
Suggested mechanism for synthesis of compound 8.

On the other hand, 3-oxobutanamide 1 was treated with hydrazine hydrate to give the pyrazole derivative 8 which confirmed ay compatible spectroscopic data and elemental analysis, as declared in Fig. 9. The suggested mechanism for preparation of compound 8 is shown in Fig. 10. The IR spectrum of compound 8 revealed disappearance of two carbonyls. ^1^H NMR spectrum showed singlet signal at *δ* = 2.10 ppm assigned to CH_3_, singlet signal referred to CH_2_ at *δ* = 2.37 ppm, OH group appeared at *δ* = 5.23 ppm, NH appeared at *δ* = 5.81 ppm, and doublet of doublet signals at *δ* = 6.43, and 6.51 ppm referred to four aromatic protons. All spectral data of the prepared compounds are represented in ESI Section as Fig. S1–S21.†

### Antibacterial assay

A disc diffusion method (Kirby–Bauer test)^32^ was used to determine the antimicrobial activity of the newly prepared compounds 2–8. Overnight bacterial cultures were diluted to 1 : 10.000 into tryptic soy broth (TSB), by measuring and adjusting the bacterial suspension OD_595_, using spectrophotometer, Spectronic™. Nutrient agar (OXOID) for bacteria was inoculated with microbial cell suspension (200 μL in 20 mL medium), and poured into sterile Petri dishes. Sterile paper discs of 6 mm diameter saturated with tested compound placed on the surface of the inoculated agar plates, the susceptibility testing following the guidelines of the National Committee for Clinical Laboratory Standards.^33,34^ Negative control was done using paper discs loaded with 20 μl of distilled water. Incubate overnight (24 h) at 37 °C. At the end of the incubation period the antibacterial activity was evaluated by measuring the inhibition zones.^35^

The result presented in Table 1, showed that compounds 6a and 6b had the best antibacterial activities close to standard drug against all types of bacteria strains tested except *Kocuria kristinae*. The compounds 4 and 7 had moderate antibacterial activity against Gram-negative bacteria *Staphylococcus haemolyticus*. Finally, compounds 2a,b, 3, 5, and 8 had not any antibacterial activities against any tested bacterial strains (Table 1). The antibacterial activity is shown in the ESI Section, as Fig. S22.†

**Table 1:** Antibacterial activity of the compounds 2–8 and standard drug against the tested bacteria by disc diffusion method (Kirby–Bauer test)^a^

Inhibition zone diameters (mm)					
Compounds (2.0 mg per disc)	Gram (−ve) bacteria	Gram (+ve) bacteria			
	*E. coli* (O157:H7)	*S. haemolyticus* (050402123720221)	*K. kristinae* (050032102000201)	*E. casseliflavus* (120200601563571)	*B. cereus* (MH 656790)
2a	−ve	−ve	−ve	−ve	−ve
2b	−ve	−ve	−ve	ND	1 ± 0.3
3	ND	−ve	−ve	−ve	ND
4	9 ± 1.2	10 ± 0.9	8 ± 0.7	11 ± 1.0	8 ± 0.9
5	3 ± 0.0	ND	−ve	ND	2 ± 0.0
6a	14 ± 1.4	19 ± 1.3	15 ± 1.6	17 ± 1.7	18 ± 0.6
6b	15 ± 0.9	20 ± 1.1	17 ± 1.3	16 ± 0.8	14 ± 0.6
7	12 ± 1.1	8 ± 0.2	11 ± 0.3	6 ± 1.0	8 ± 1.0
8	−ve	2 ± 0.3	ND	1 ± 0.0	−ve
**Standard**	**Levofloxacin (20 mg per disc)**				
St. result	17 ± 0.5	21 ± 0.7	20 ± 0.3	20 ± 1.0	16 ± 0.7

a Abbreviations: −ve: no activity, ND: not determined. Values are mean inhibition zone diameter (mm) ± standard deviation of three replicates.

MIC values showed promising results for most tested compounds as shown in Table 2. Within the tested bacteria, the MIC values were ranged from 0.5 to 2 mg mL^−1^. Interestingly, both Gram-negative and Gram-positive bacteria were sensitive to the compounds. The antimicrobial activity can be modulated by the presence of pyrimidine moiety, amide linkage, and phenolic substrate.

**Table 2:** MIC values of compounds 2–8

MIC^a^ (mg mL^−1^)					
Compounds (2.0 mg per disc)	Gram (−ve) bacteria	Gram (+ve) bacteria			
	*E. coli*	*S. haemolyticus*	*K. kristinae*	*E. casseliflavus*	*B. cereus*
2a	−ve	−ve	−ve	−ve	−ve
2b	−ve	−ve	−ve	ND	1 ± 0.3
3	ND	−ve	−ve	−ve	ND
4	1.6 ± 0.2	1.4 ± 0.3	1.5 ± 0.3	1.5 ± 0.1	1.6 ± 0.2
5	2.0 ± 0.0	ND	−ve	ND	2.0 ± 0.0
6a	0.9 ± 0.2	0.6 ± 0.3	0.6 ± 0.2	0.7 ± 0.2	0.5 ± 0.1
6b	0.5 ± 0.05	0.3 ± 0.1	0.5 ± 0.1	0.4 ± 0.1	0.4 ± 0.05
7	0.9 ± 0.1	1.2 ± 0.2	1.1 ± 0.3	1.8 ± 0.1	1.5 ± 0.1
8	−ve	2.0 ± 0.3	ND	1.0 ± 0.0	−ve
**Standard**	**Levofloxacin (20 mg per disc)**				
St. result	1.6 ± 0.4	2.0 ± 0.2	2.0 ± 0.3	2.5 ± 0.2	2.2 ± 0.3

a MIC: minimum inhibitory concentration (mg mL^−1^).

### Computational study

To investigate the affinity and orientation of the newly synthesized molecules against the active site of the target enzyme, an *in silico* study was performed.^36,37^ In the present study, bacterial flavoHB enzyme (PDB: 3OZU)^38^ has been selected as therapeutic target for identification of antibacterial drug candidates through molecular docking technique. In addition, levofloxacin was selected as a standard drug to compare its binding affinity with those of the docked compounds.

The compounds 6a,b with the strong antibacterial activities, exhibited the highest binding affinity to the target enzyme with binding energies Δ*G*s (−10.4 and −10.0 kcal mol^−1^), respectively, higher than of the standard drug (Table 3). The compound 6a engaged to the binding site of the target through three hydrogen bond interactions with the residues Tyr29, and Ala130 at the distances of 2.98, 3.01, and 2.98 Å. While the derivative 6b docked to the target though one hydrogen bond with Ala130 at 2.95 Å. Compound 2a interacted with Glu226 of the target enzyme at 2.40 Å, while, derivative 2b formed two hydrogen bonds and arene–cation interaction with the residues Lys224, Tyr235, and Arg206. On the other hand, compound 3 formed arene-stacked interactions such as arene–arene and arene–cation with Phe43 and Val98 at 5.15, and 3.49 Å, respectively (Table 3). The compound 5 exhibited four HBs and two arene–cation interactions with the amino acid residues Lys84, Gln207, Lys224, Tyr235, and Arg206. Compound 7 with the binding energy Δ*G* −6.3 kcal mol^−1^, showed one HB interactions with the residue Asn80. Finally, the docked molecule 8 formed two HB interactions with His392, and Ile391 at the distances 2.96, and 2.34 Å, respectively (Table 2).

**Table 3:** The binding energies of the prepared molecules and their molecular interactions with the active site of the target enzyme

	2D structure	B.E.^a^ (kcal mol^−1^)	Docked complex (amino acid–ligand) interactions	Distance (Å)
2a	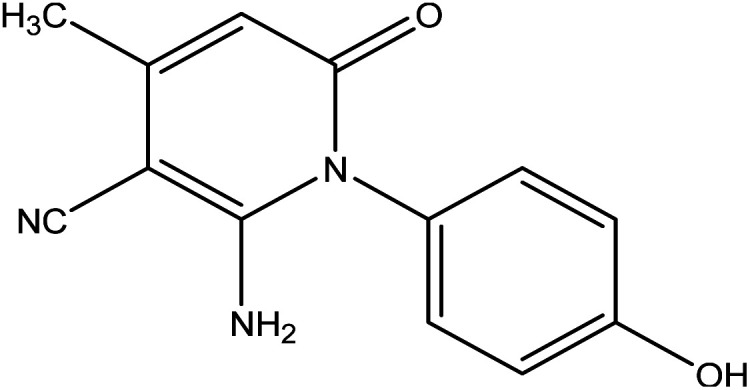	−7.0	**H-Bond**	
			Glu226:OE2—compound 2a	2.40
2b	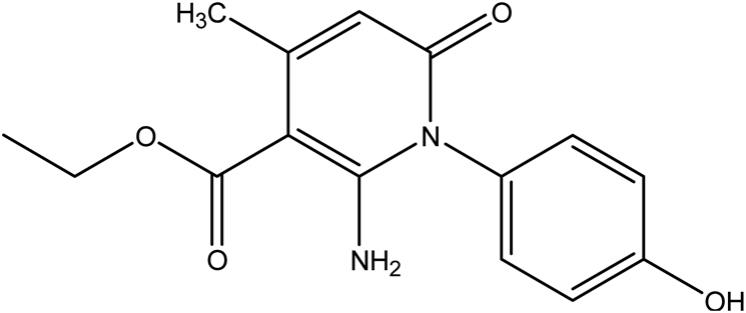	−7.1	**H-Bond**	
			Lys224:N—compound 2b	3.00
			Tyr235:N—compound 2b	2.96
			**Arene–cation**	
			Arg206:NH2—compound 2b	5.13
3	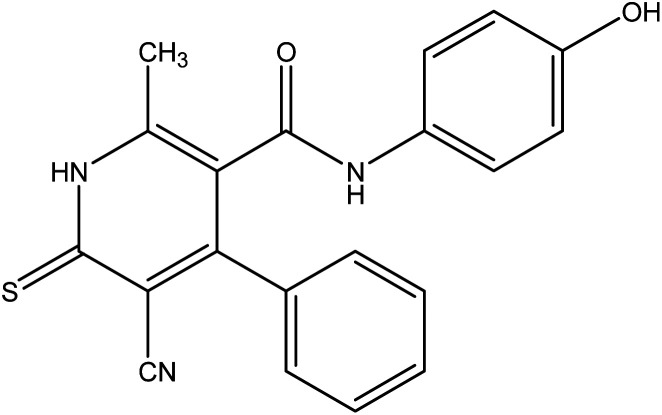	−9.1	**Arene–arene**	
			Phe43—compound 3	5.15
			**Arene–cation**	
			Val98:CG2—compound 3	3.49
4	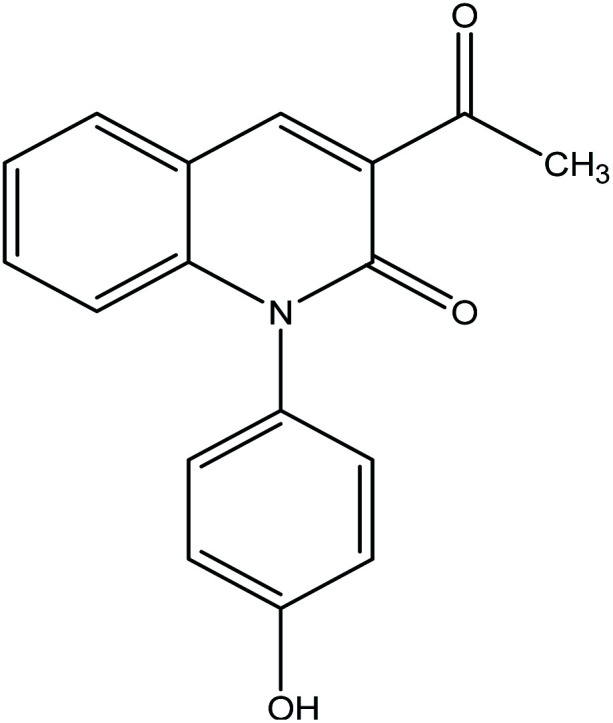	−8.1	**H-Bond**	
			Tyr126:OH—compound 4	2.96
5	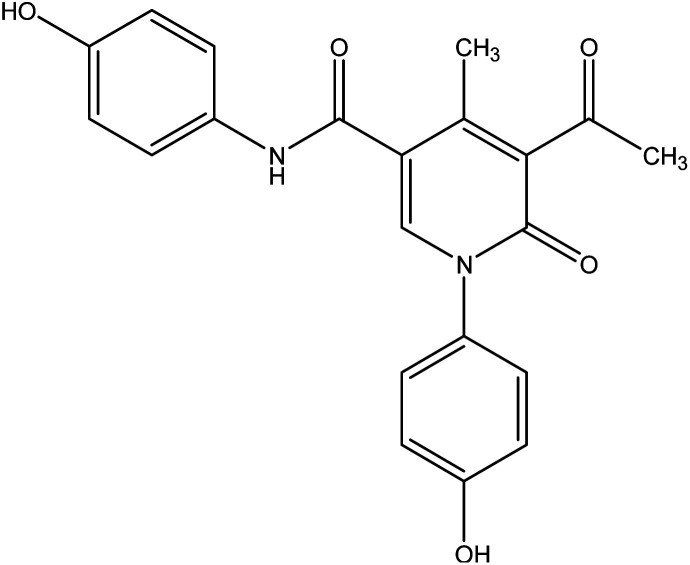	−9.6	**H-Bond**	
			Lys84:NZ—compound 5	2.89
			Gln207:N—compound 5	2.98
			Lys224:N—compound 5	3.00
			Tyr235:N—compound 5	2.96
			**Arene–cation**	
			Arg206:NH1—compound 5	4.42
			Arg206:NH2—compound 5	5.98
6a	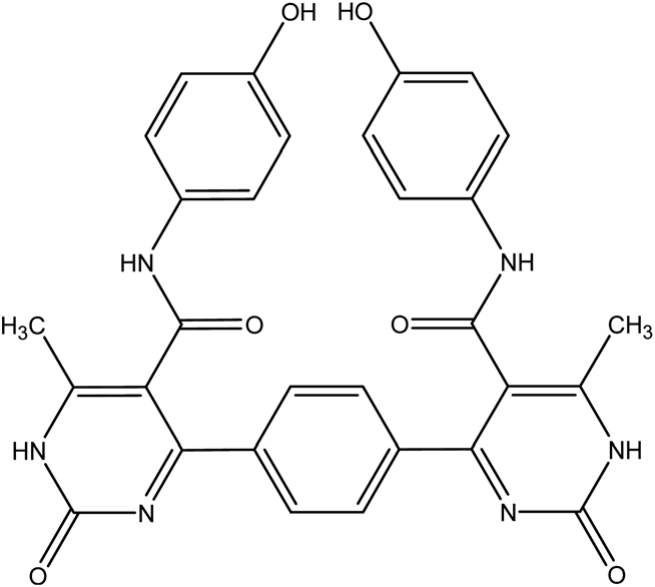	−10.4	**H-Bond**	
			Tyr29:OH—compound 6a	2.98
			Tyr29:OH—compound 6a	3.01
			Ala130:N—compound 6a	2.98
6b	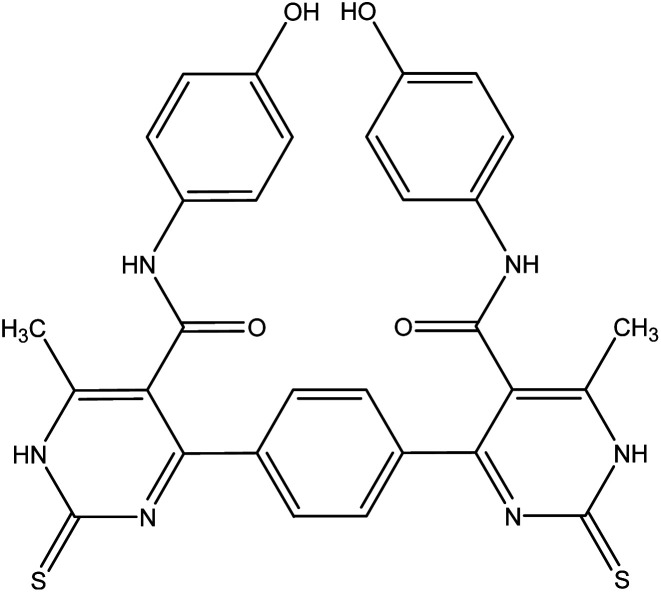	−10.0	**H-Bond**	
			Ala130:N—compound 6b	2.95
7	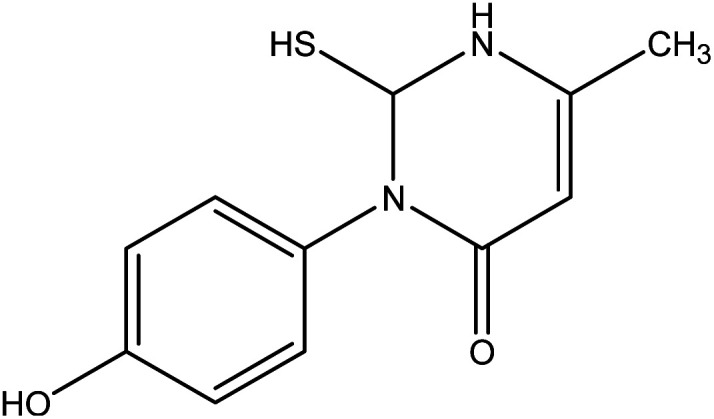	−6.3	**H-Bond**	
			Asn80:ND2—compound 7	3.08
8	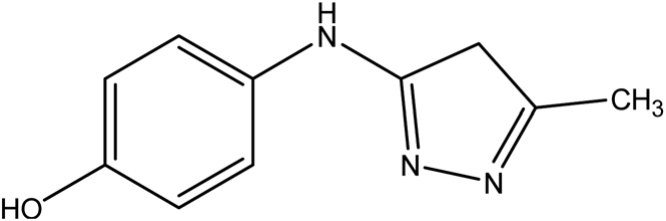	−6.9	**H-Bond**	
			His392:ND1—compound 8	2.96
			Ile391:O—compound 8	2.34
Standard drug	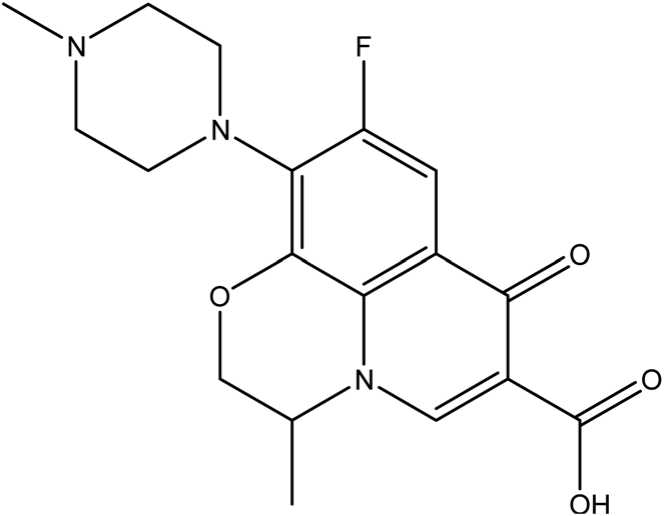	−7.9	**H-Bond**	
			Tyr393:N—standard drug	3.18
			Glu388:OE1—standard drug	2.40
			**Arene–arene**	
			Tyr393—standard drug	4.55

a B.E. binding energy.

On the other hand, the standard drug, levofloxacin exhibited binding energy of −7.9 kcal mol^−1^, and showed two HBs and one arene–arene interactions with the residues Tyr393 and Glu388 at the distances 3.18, 2.40, and 4.55 Å, respectively (Table 3).

**Fig. 11:**
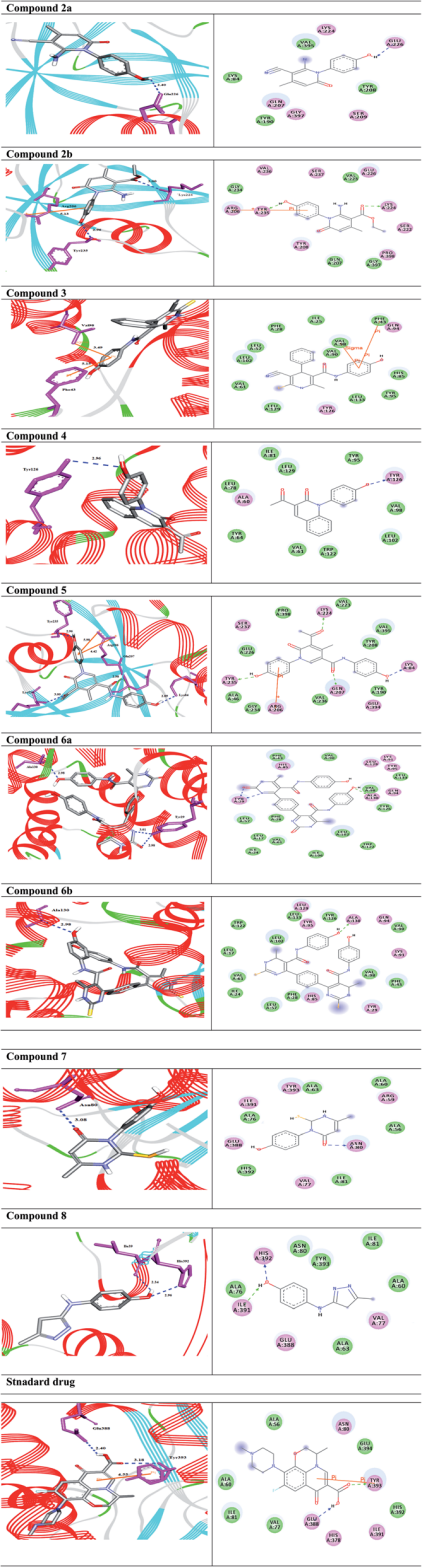
(Left side) 3D, and (Right side) 2D representations of the docked complexes (compounds and standard drug with the target enzyme). (Left side); H-bonds are represented in blue dotted lines while arene-interactions are shown in orange lines. (Right side); the amino acids are shown in 3 letter code, H-bonds are declared in blue and green dotted lines, while arene-interactions are shown in orange lines.

The 2D and 3D representations of the intermolecular interactions between all docked compounds and standard drug with the active site residues of the target enzyme flavoHB are shown in Fig. 11.

Furthermore, the ADMET and drug-likeness results showed interesting values for the compounds (Table 4). All the prepared compounds have molecular weights in the expected range of 130–725 g mol^−1^. In addition, they have better human intestinal absorption (HIA^+^) score in the range of 66.31–100%, which verified that they could be better absorbed by the human intestine. Further, HBA (H-bond acceptors) and HBD (H-bond donors) satisfy Lipinski's rule of five and were found to be in the acceptable range. The rotatable bonds in the compounds are in limits (1–6). All ligand molecules except 4 and 8 does not pass the blood–brain barrier that confirming their good CNS safety profile. Finally, all the compounds displayed negative carcinogenicity tests. According to Lipinski's rule of five which states that a molecule violating more than one, it is less likely to be a drug. Therefore, the findings confirmed that the ligand molecules 6a and 6b that containing pyrimidine moiety, amide linkage, and phenolic substrate could be used as potent and safe flavoHB inhibitors as promising antibacterial therapeutics.

**Table 4:** Physicochemical and drug-likeness properties of the synthesized compounds 2–8 and the standard drug^a^

	Compound 2a	Compound 2b	Compound 3	Compound 4	Compound 5	Compound 6a	Compound 6b	Compound 7	Compound 8	Standard drug
Molecular weight (g mol^−1^)	241.25	288.30	361.43	279.30	378.38	564.56	596.69	236.30	189.22	361.37
BBB permeant	No	No	No	Yes	No	No	No	No	Yes	No
% Human intestinal absorption (HIA^+^)	99.36	84.63	89.53	99.66	93.36	93.76	66.31	92.02	100.00	95.45
log *p*	1.13	1.59	2.91	2.30	1.02	2.28	2.96	1.81	1.66	0.98
HBA	5	6	5	4	7	12	10	4	4	7
HBD	3	3	3	1	3	6	6	2	2	1
N rotatable	1	4	3	2	4	6	6	1	2	2
N violations	0	0	0	0	0	1	1	0	0	0
Volume (A^3^)	212.53	257.00	312.53	247.36	330.74	481.23	498.99	203.85	173.51	311.15
AMES toxicity	Toxic	Nontoxic	Nontoxic	Nontoxic	Nontoxic	Nontoxic	Nontoxic	Nontoxic	Toxic	Nontoxic
Carcinogenicity	Noncarcinogenic	Noncarcinogenic	Noncarcinogenic	Noncarcinogenic	Noncarcinogenic	Noncarcinogenic	Noncarcinogenic	Noncarcinogenic	Noncarcinogenic	Noncarcinogenic
GI absorption	High	High	High	High	High	Low	Low	High	High	High
Bioavailability score	0.55	0.55	0.55	0.55	0.55	0.17	0.17	0.55	0.55	0.55

a Abbreviations and acceptable ranges are as follows; HBA, number of hydrogen bond acceptors (2.0–20.0); HBD, number of hydrogen bond donors (0.0–6.0); log *p*, logarithm of partition coefficient between *n*-octanol and water (<5); N rotatable, number of rotatable bonds (≤10); TPSA, topological polar surface area (≤140); Mol wt.: (130–725); % (HIA^+^) human oral absorption: >80% high, <25% low; volume: (500–2000).

## Materials and methods

### Chemistry

All melting points were determined on an electrothermal apparatus and are uncorrected. IR spectra were recorded (KBr discs) on a Shimadzu FT-IR 8201 PC spectrophotometer. ^1^H-NMR and ^13^C-NMR spectra (400 MHz) were recorded in DMSO (CD3)_2_SO solution on a BRUKER500 FT-NMR system spectrometer, and chemical shifts are expressed in ppm units using TMS as an internal reference. Mass spectrum was recorded on a GC-MS QP1000 EX Shimadzu. Elemental analyses were carried out at the Microanalytical Center of Cairo University, Egypt.

#### General procedure for preparation of compounds 2a,b

A mixture of compound 1 (1.93 g, 10 mmol) and malononitrile (0.66 g, 10 mmol) or ethyl cyanoacetate (1.13 g, 10 mmol) was refluxed in ethanol containing few drops of piperidine for 5 h. The reaction mixture was left to cool, then poured onto ice/water containing few drops of conc. HCl. The formed solid product was collected by filtration and recrystallized from ethanol to afford pyridinone derivative 2a,b.

##### 1-(4-Hydroxyphenyl)-2-imino-4-methyl-6-oxo-1,2,5,6-tetrahydropyridine-3-carbonitrile 2a

Compound 2a was obtained as brownish crystals, yield (60%), mp = 110–112 °C, IR (KBr) *ν* cm^−1^; 3330 (OH), 3207, 2978 (NH_2_), 2194 (CN), 1633 (C=O). ^1^H NMR (DMSO-*d*_6_) (*δ* ppm); *δ*_H_ = 2.40 (s, 3H, CH_3_), 3.73 (s, 2H, NH_2_), 6.46–7.02 (m, 4H, Ar-H), 7.64 (s, 1H, CH-pyridine), 9.33 (hump, 1H, OH). Anal. calcd for C_13_H_11_N_3_O_2_ (241.25): C, 64.72; H, 4.60; N, 17.42%. Found C, 64.45; H, 4.77; N, 17.25.

##### Ethyl-2-amino-1-(4-hydroxyphenyl)-4-methyl-6-oxo-1,6-di-hydropyridine-3-carboxylate 2b

Compound 2b was obtained as brown crystals (60%); mp = 129–131 °C; IR (KBr) *ν* cm^−1^; 3330 (OH), 3207, 2978 (NH_2_), 1722 (C=O), 1636 (C=O-amide). ^1^H NMR (DMSO-*d*_6_) (*δ* ppm); *δ*_H_ = 1.12 (t, 3H, CH_3_, *J* = 4 Hz), 2.28 (s, 3H, CH_3_), 4.04 (q, 2H, CH_2_), 5.14 (s, 2H, NH_2_), 6.64–7.92 (m, 4H, Ar-H), 8.12 (s, 1H, CH-pyridine), 10.32 (s, 1H, OH). Anal. calcd for C_15_H_16_N_2_O_4_ (288.30): C, 62.49; H, 5.59; N, 9.72%. Found C, 62.85; H, 5.70; N, 9.40.

##### 4-(4-Chlorophenyl)-5-cyano-*N*-(4-hydroxyphenyl)-2-methyl-6-thioxo-1,6-dihydro pyridine-3-carboxamide 3

A mixture of 1 (1.93 g, 10 mmol), and 2-cyano-3-phenylprop-2-enethioamide (1.88 g, 10 mmol), was refluxed in ethanol containing drops of piperidine for 5 h, The reaction mixture was left to cool, then poured onto ice/water containing few drops of conc. HCl. The formed solid product was collected by filtration and recrystallized from ethanol to afford pyridine thiol derivative 3. Yield (61%), color: dark red crystals, mp = 121–123 °C; IR (KBr) *ν* cm^−1^; 3298 (OH), 3193, 3180 (2NH), 2216 (CN), 1631 (C=O). ^1^H NMR (DMSO-*d*_6_) (*δ* ppm); *δ*_H_ = 2.38 (s, 3H, CH_3_), 3.93 (s, 1H, NH), 6.45–7.57 (m, 8H, Ar-H), 9.46 (s, 1H, NH-Ph), 9.83 (s, 1H, OH). Anal. calcd for C_20_H_14_ClN_3_O_2_S (395.86): C, 60.68; H, 3.56; Cl, 8.96; N, 10.61; S, 8.10 Found C, 60.71; H, 3.45; Cl, 8.91; N, 10.50; S, 8.27.

##### 3-Acetyl-1-(4-hydroxyphenyl)quinolin-2(1H)-one 4

A mixture of compound 1 (1.93 g, 10 mmol), and salicylaldehyde (1.22 g, 10 mmol), was refluxed in ethanol containing drops of piperidine for 5 h. The reaction mixture was left to cool, then poured onto ice/water containing few drops of conc. HCl. The formed solid product was collected by filtration and recrystallized from ethyl acetate to yield 3-acetyl-1-(4-hydroxyphenyl) quinolin-2(1H)-one 4. Yield (96%), color: brown crystals; mp = 105–107 °C; IR (KBr) *ν* cm^−1^; 3278 (OH), 1707 (C=O-acetyl), 1662 (C=O-amide). ^1^H NMR (DMSO-*d*_6_) (*δ* ppm); *δ*_H_ = 2.38 (s, 3H, CH_3_), 6.85–7.37 (m, 8H, Ar-H), 8.88 (s, 1H, CH-pyridine), 9.55 (s, 1H, OH). Anal. calcd for C_17_H_13_NO_3_ (279.29): C, 73.11; H, 4.69; N, 5.02. Found C, 73.56; H, 4.65; N, 4.80.

##### 5-Acetyl-*N*,1-bis(4-hydroxyphenyl)-4-methyl-6-oxo-1,6-dihydropyridine-3-carboxamide 5

A mixture of 3-oxobutanamide 1 (1.93 g, 10 mmol), and DMF-DMA (1.19 g, 10 mmol), was refluxed in *p*-xylene for 4 h, the solid product formed was filtered off and recrystallized from ethanol to furnish pyridinone derivative 5. Yield (62%), color: grey crystals, mp = 190–192 °C, IR (KBr) *ν* cm^−1^; 3430 (OH) 3231 (NH), 1710 (C=O), 1650 (C=O). ^1^H NMR (DMSO-*d*_6_) (*δ* ppm); 1.91 (s, 3H, CH_3_), 2.18 (s, 3H, CH_3_), 6.69–7.21 (m, 8H, Ar-H), 8.21 (s, 1H, CH-pyridine), 9.50 (hump, 2H, 2OH), 11.46 (s, 1H, NH). ^13^C NMR *δ*_C_ = 19.73 (CH_3_), 21.47 (CH_3_), 55.72, 114.48, 115.91, 116.90, 120.70, 121.64, 129.54, 129.75, 132.08, 152.80, 158.64, 164.25 (C=O), 176.37 (C=O), 196.46 (C=O). MS (relative intensity) *m*/*z*: 377 (M^−1^, 5%), 245 (10%), 165 (17%), 105 (60%), 44 (100%). Anal. calcd for C_21_H_18_N_2_O_5_ (378.38): C, 66.66; H, 4.79; N, 7.40%. Found C, 66.54; H, 4.60; N, 7.37.

#### General procedure for preparation of compounds 6a,b

A mixture of three component one pot; 3-oxobutanamide 1 (3.86 g, 20 mmol), terephthalaldehyde (1.35 g, 10 mmol), and urea (1.2 g, 20 mmol) or thiourea (1.52 g, 20 mmol) was refluxed in ethanol containing drops of hydrochloric acid for 6 h, the solid product formed was filtered off and crystallized from benzene to give pyrimidinone 6a and thiol derivatives 6b, respectively.

##### 4,4′-(1,4-Phenylene)bis(*N*-(4-hydroxyphenyl))-6-methyl-2-oxo-1,2-dihydropyrimidine-5-carboxamide 6a

Compound 6a was obtained as dark yellow crystals. Yield (50%), mp. = 300–302 °C, IR (KBr) *ν* cm^−1^; 3301 (2OH), 3167 (2NH), 3104 (2NH), 1662 (C=O amide). ^1^H NMR (DMSO-*d*_6_) (*δ* ppm); *δ*_H_ = 2.28 (s, 6H, CH_3_), 5.14 (s, 2H, OH), 6.64–8.12 (m, 12H, Ar-H), 9.61 (s, 2H, NH-amide), 10.32 (s, 2H, NH-pyrimidine). ^13^C NMR *δ*_C_ = 17.62, 101.20, 115.40, 127.05, 127.66, 130.41, 143.47, 145.44, 165.57, 174.80. Dept. 135 NMR *δ*_C_ = (+) 17.61, 115.39, 127.05, 127.66, 130.40, 130.44. Anal. calcd for C_30_H_24_N_6_O_6_ (564.55): C, 63.82; H, 4.28; N, 14.89%. Found C, 63.60; H, 4.19; N, 14.90.

##### 4,4′-(1,4-Phenylene)bis(*N*-(4-hydroxyphenyl))-6-methyl-2-thioxo-1,2-dihydropyrimidine-5-carboxamide 6b

Compound 6b was obtained as yellow crystals. Yield (64%), mp = 118–120 °C, yield (64%), color: yellow crystals; mp = 118–120 °C; IR (KBr) *ν* cm^−1^; 3221 (OH), 3186 (b, 4NH), 1686 (C=O). ^1^H NMR (DMSO-*d*_6_) (*δ* ppm); *δ*_H_ = 2.33 (s, 6H, CH_3_), 5.29 (s, 2H, OH), 6.66–8.12 (m, 12H, Ar-H), 8.12 (s, 2H, NH-amide), 10.00 (s, 2H, NH-pyrimidine). ^13^C NMR *δ*_C_ = 17.65, 100.69, 115.45, 122.23, 127.64, 130.34, 136.16, 145.98, 150.12, 165.45, 175.09, 192.19. Dept. 135 NMR *δ*_C_ = (+) 17.66, 54.56, 115.45, 122.23, 127.59, 130.34, 192.91. MS (relative intensity) *m*/*z*: 596 (M, 0.15%), 368 (M, 0.78%), 304 (M, 9.56%), 275 (M, 10.31%), 134 (M, 29.91%), 105 (M, 47.59%), 57 (M, 100%). Anal. calcd for C_30_H_24_N_6_O_4_S_2_ (596.68): C, 60.39; H, 4.05; N, 14.08; S, 10.75%. Found C, 60.70; H, 4.30; N, 14.20; S, 10.70%.

##### 3-(4-Hydroxy-phenyl)-2-mercapto-6-methyl-2,3-dihydro-1*H*-pyrimidin-4-one 7

A mixture of 3-oxobutanamide 1 (1.93 g, 10 mmol), and ethyl isothiocyanate (0.87 g, 10 mmol), was refluxed in ethanol containing drops of piperidine for 6 h, the formed sticky product precipitated by drops of HCl, then collected by filtration and recrystallized from ethanol to afford pyrimidine derivative 7. Yield (73%), color: yellow crystals, mp. = 220–222 °C, IR (KBr) *ν* cm^−1^; 3300 (OH), 3163 (NH), 1660 (C=O amide). ^1^H NMR (DMSO-*d*_6_) (*δ* ppm); *δ*_H_ = 1.10 (s, 3H, CH_3_), 6.89–7.27 (dd, 4H, Ar-H, *J* = 14 Hz), 9.32 (s, 1H, CH-pyrimidine), 9.77 (s, 1H, SH), 10.15 (s, 1H, OH). ^13^C NMR *δ*_C_ = 14.82, 104.36, 116.19, 116.51, 122.93, 124.74, 126.39, 155.18, 157.61, 165.72. MS (relative intensity) *m*/*z*: 236 (M^−1^, 56%), 163 (50%), 151 (49%), 109 (100%). Anal. calcd for C_30_H_24_N_6_O_6_ (236.06): C, 55.91; H, 5.12; N, 11.86; S, 13.57%. Found C, 55.95; H, 5.40; N, 11.90; S, 13.51.

##### 4-((5-Methyl-4*H*-pyrazol-3-yl)amino)phenol 8

A mixture of compound 1 (1.93 g, 10 mmol), and hydrazine hydrate (0.46 g, 10 mmol), was refluxed in ethanol containing drops of piperidine for 6 h, then the formed solid product was collected by filtration and recrystallized from benzene to yield pyrazole derivative 8. Yield (78%), color: silver crystals, mp = 144–146 °C; IR (KBr) *ν* cm^−1^; 3337 and 3278 (OH, NH). ^1^H NMR (DMSO-*d*_6_) (*δ* ppm); *δ*_H_ = 2.10 (s, 3H, CH_3_), 2.37 (s, 2H, CH_2_), 5.23 (s, 1H, OH), 5.81 (s, 1H, NH), 6.43–6.51 (dd, 4H, Ar-H, *J* = 24 Hz). Anal. calcd for C_10_H_11_N_3_O (189.21): C, 63.48; H, 5.86; N, 22.21%. Found C, 63.50; H, 5.99; N, 22.40.

### Biological assessment

Compounds 2–8 (2.0 mg per disc), in addition to the standard drug levofloxacin (20 mg per disc) (ready for use discs from Bioanalysis company Turkey, https://www.bioanalysis.com) and control dimethyl sulphoxide (DMSO) were tested *in vitro* against pathogenic bacterial strains *Escherichia coli* (O157:H7), *Staphylococcus haemolyticus* (050402123720221), *Kocuria kristinae* (050032102000201), *Enterococcus casseliflavus* (120200601563571), and *Bacillus cereus* (MH 656790) (identified in Al-Azhar University, center of mycology and the regional biotechnology – by using BIOMERIEUX VITEK2 SYSTEM-bio number), as shown in the ESI as Fig. S23.†

### Determination of the minimum inhibitory concentration (MIC)

The determination of MIC was assayed as described earlier by Salem *et al.*^39^ The freshly prepared cultures of both Gram-negative bacteria and/or Gram-positive bacteria were adjusted to OD_595_ of 0.001. 100 μL of each isolate culture was put into sterilized 96-well plates. Then 20 μL of the screened compounds (2 mg mL^−1^) (serial dilutions of 10^−1^ to 10^−10^ were used, 8 replicates were made for each dilution into complete raw of the 96-well plate). In addition, levofloxacin (20 mg per disc) was used as reference drug. The un-inoculated media tested as negative control, after 24 h incubation at 37 °C. MIC was determined by the addition of 40 μL of *p*-iodonitrotetrazolium violet chloride (INT) (0.2 μg mL^−1^, Sigma-Aldrich) to the plates and re-incubated at 37 °C for 30 min., the lowest concentration which banned color change is the MIC.^40^

### *In silico* docking approach

Molecular docking study^41,42^ of ligand molecules was carried out to evaluate their binding geometries with the target enzyme flavoHB. To understand the binding mode of interactions of newly synthesized compounds with the target, the crystallographic structure of the enzyme was downloaded from the Protein Data Bank (PDB: 3OZU).^38^ Additionally, the 2D structures of the ligand molecules were sketched using ChemDraw 16, and converted to SDF format using Open Babel GUI.^43^ The docking approach was carried out using PyRx-virtual screening tool.^44^ To obtain the 2D and 3D intermolecular representations of the docked compounds, the discovery studio 3.5 was employed. Moreover, *in silico* ADMET and drug-likeness prediction of the molecules were performed using admetSAR, mol inspiration, and SwissADME free accessible tools.

## Conclusion

Novel series of nitrogen-based heterocycles attached to *p*-phenolic unit was synthesized. *In vitro* study showed that compounds 6a and 6b had appreciable antibacterial activities close to standard drug against all types of bacteria strains tested. Consequently, *in silico* study revealed that compounds 6a and 6b showed good binding energies against the target enzyme. Overall; the findings exhibited that compounds 6a and 6b represent promising starting points for the development of novel antibacterial inhibitors.

## Data availability

The original contributions presented in the study are included in the article/ESI;† further inquiries can be directed to the corresponding author.

## Author contributions

All authors made a significant contribution to the work reported, whether that is in the conception, study design, execution, acquisition of data, analysis, and interpretation, or in all these areas; took part in drafting, revising or critically reviewing the article; gave final approval of the version to be published; have agreed on the journal to which the article has been submitted; and agree to be accountable for all aspects of the work.

## Conflicts of interest

The authors declare no conflict of interest.

## Supplementary Material

RA-012-D2RA01794F-s001
