# Supplementary material for: Synthesis, characterization, in silico molecular docking, and antibacterial activities of some new nitrogen-heterocyclic analogues based on a p-phenolic unit
Source: RSC Adv. 2022 Apr 26;12(20):12607–21. doi: 10.1039/d2ra01794f (PMC9041306; doi:10.1039/d2ra01794f)
Supplement: RA-012-D2RA01794F-s001 [file RA-012-D2RA01794F-s001.pdf]

Electronic Supplementary Material (ESI) for RSC Advances.
This journal is © The Royal Society of Chemistry 2022

# Synthesis, Characterization, *In silico* Molecular Docking, and Antibacterial Activities of Some New Nitrogen-heterocyclic Analogues Based *p*-Phenolic Unit

Abdel Haleem M. Hussein[1], Abu-Bakr A. El-Adasy[1], Ahmed M. El-Saghier[2], M. Olish[1], and Aboubakr H. Abdelmonsef[3]*

[1]Chemistry Department, Faculty of Science, Al-Azhar University, Assiut, Egypt

[2] Chemistry Department, Faculty of Science, Sohag University, 82524 Sohag, Egypt

[3] Chemistry Department, Faculty of Science, South Valley University, 83523 Qena, Egypt

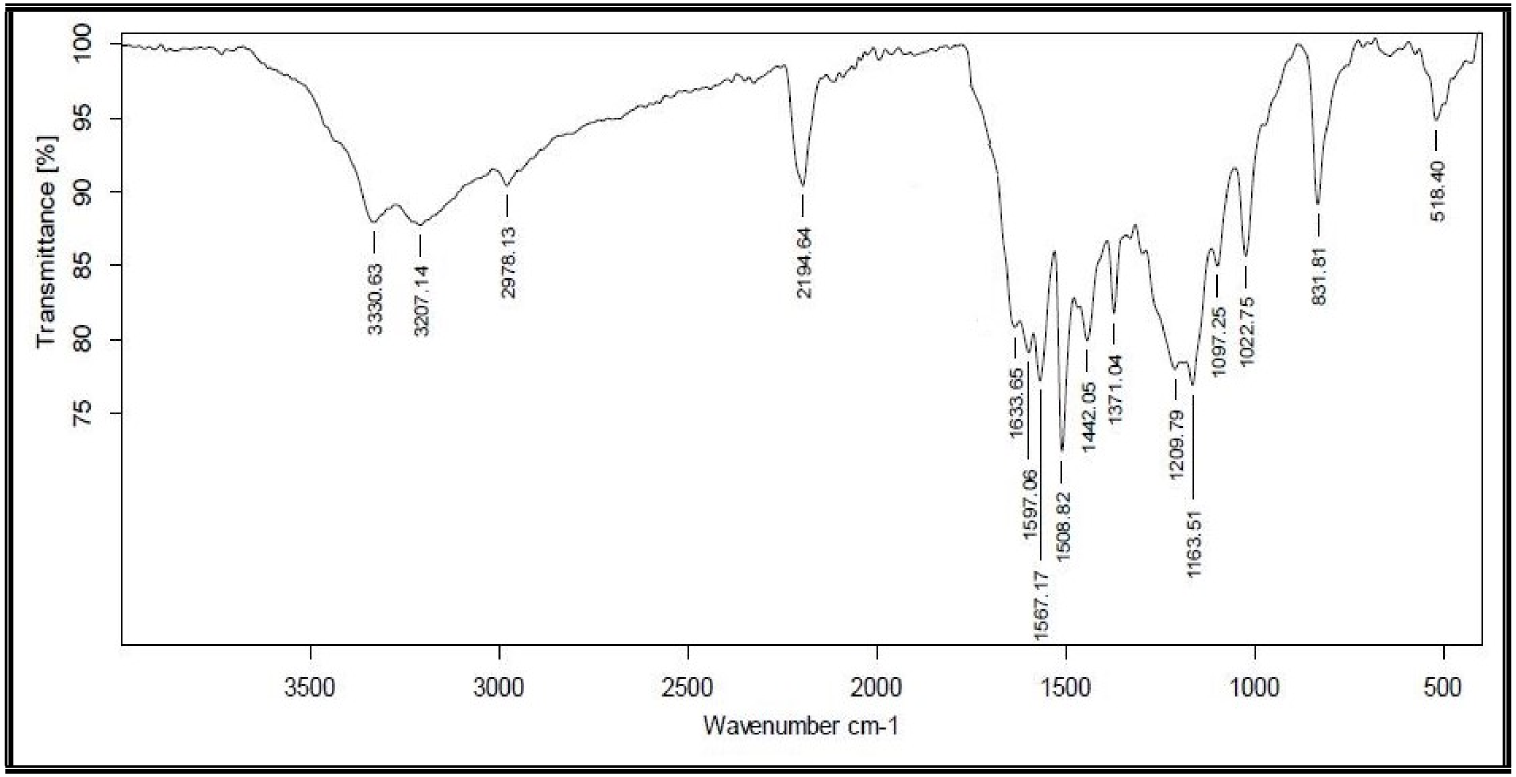


**Figure S1.** IR Spectrum of Compound (2a)

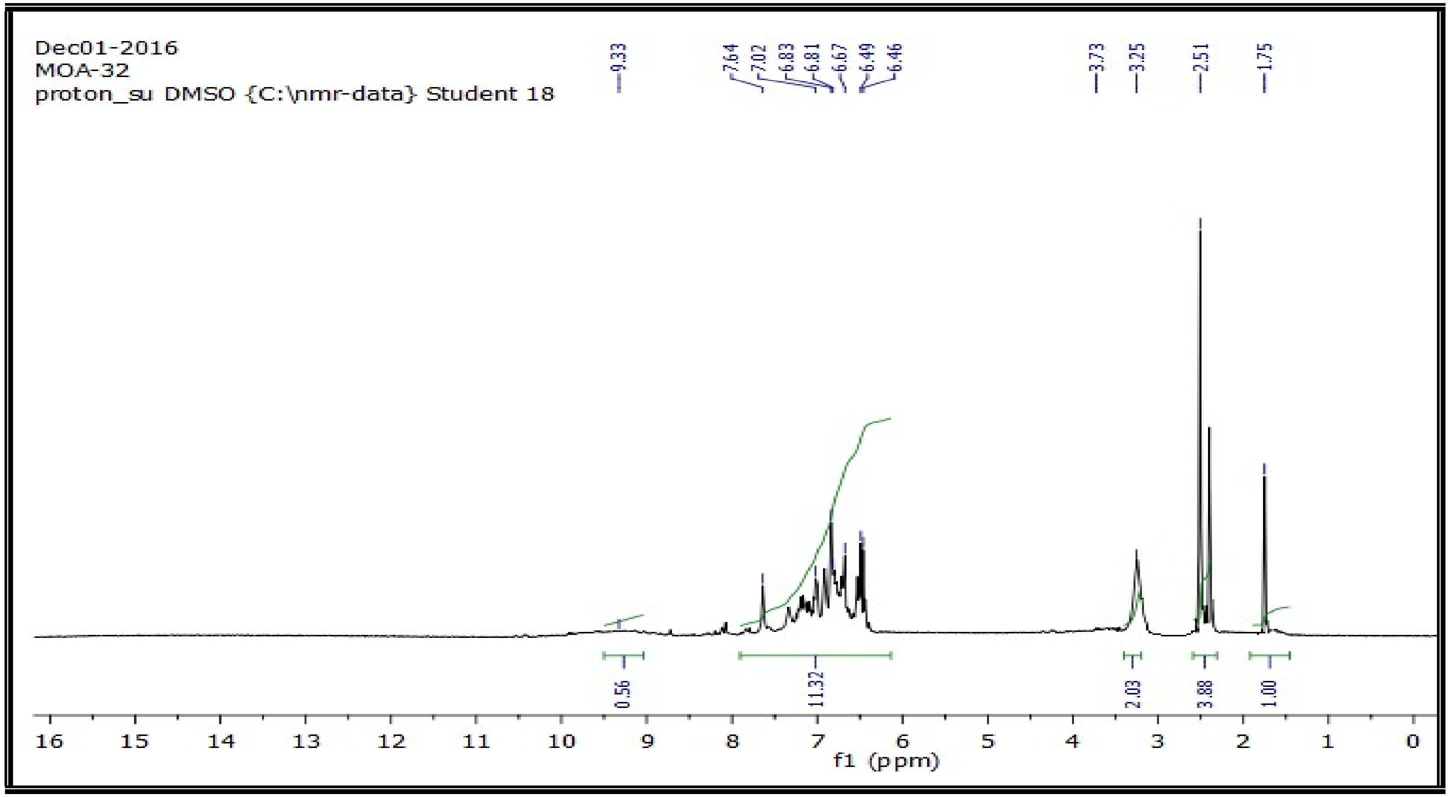


**Figure S2.** $^{1}$H NMR Spectrum of Compound (2a)

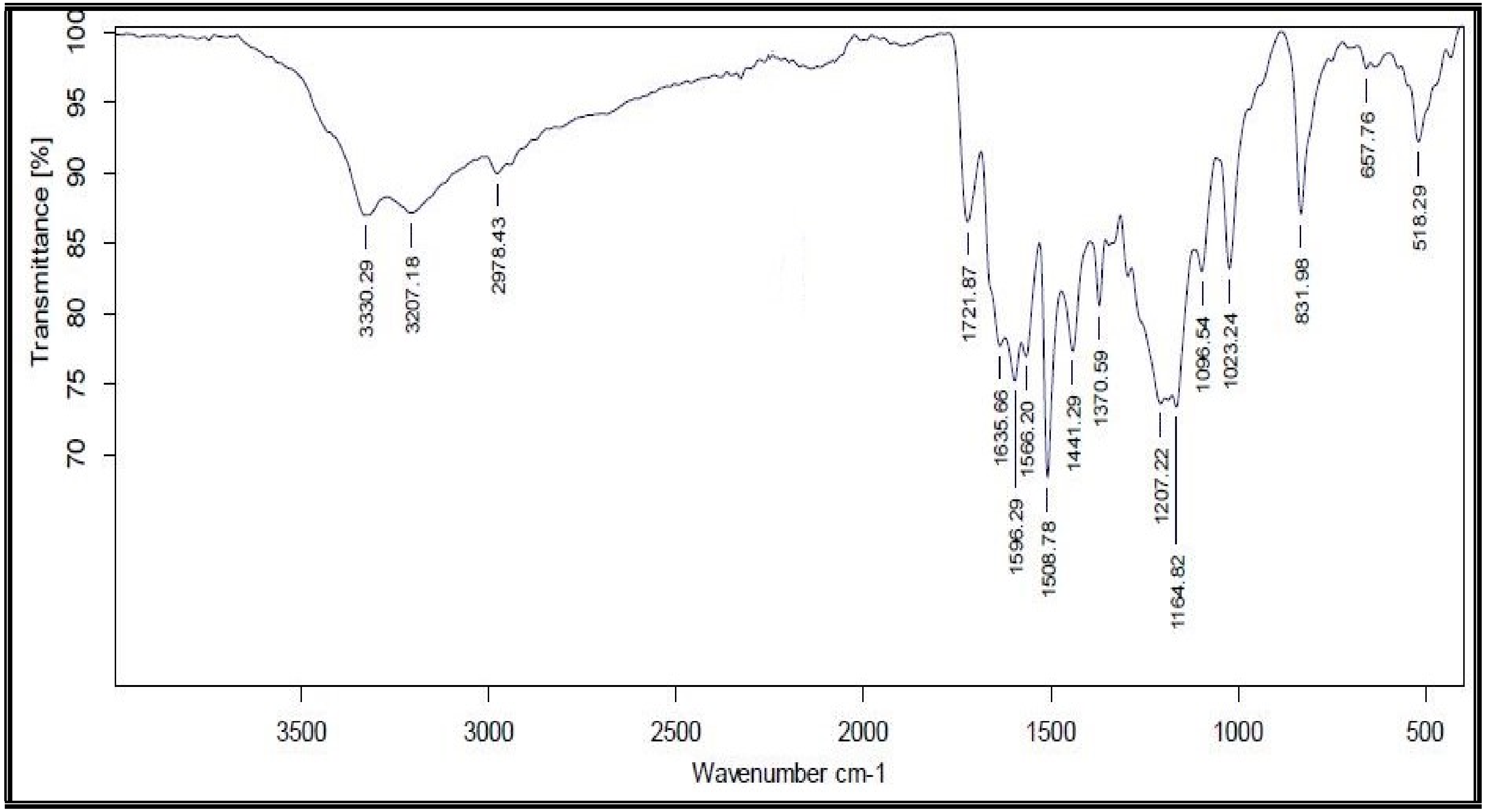


**Figure S3.** IR Spectrum of Compound (2b)

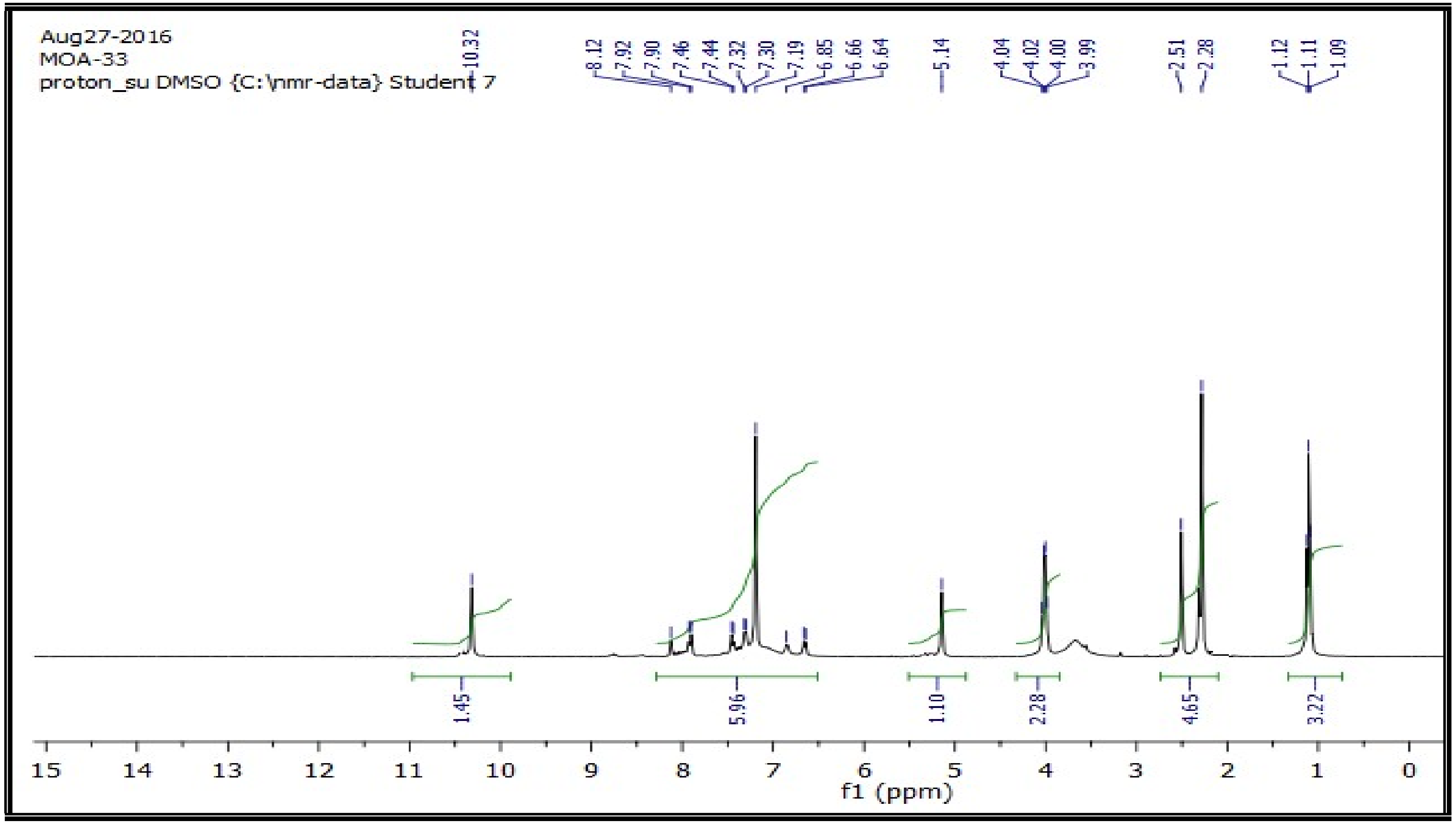


**Figure S4.** $^{1}$H NMR Spectrum of Compound (2b)

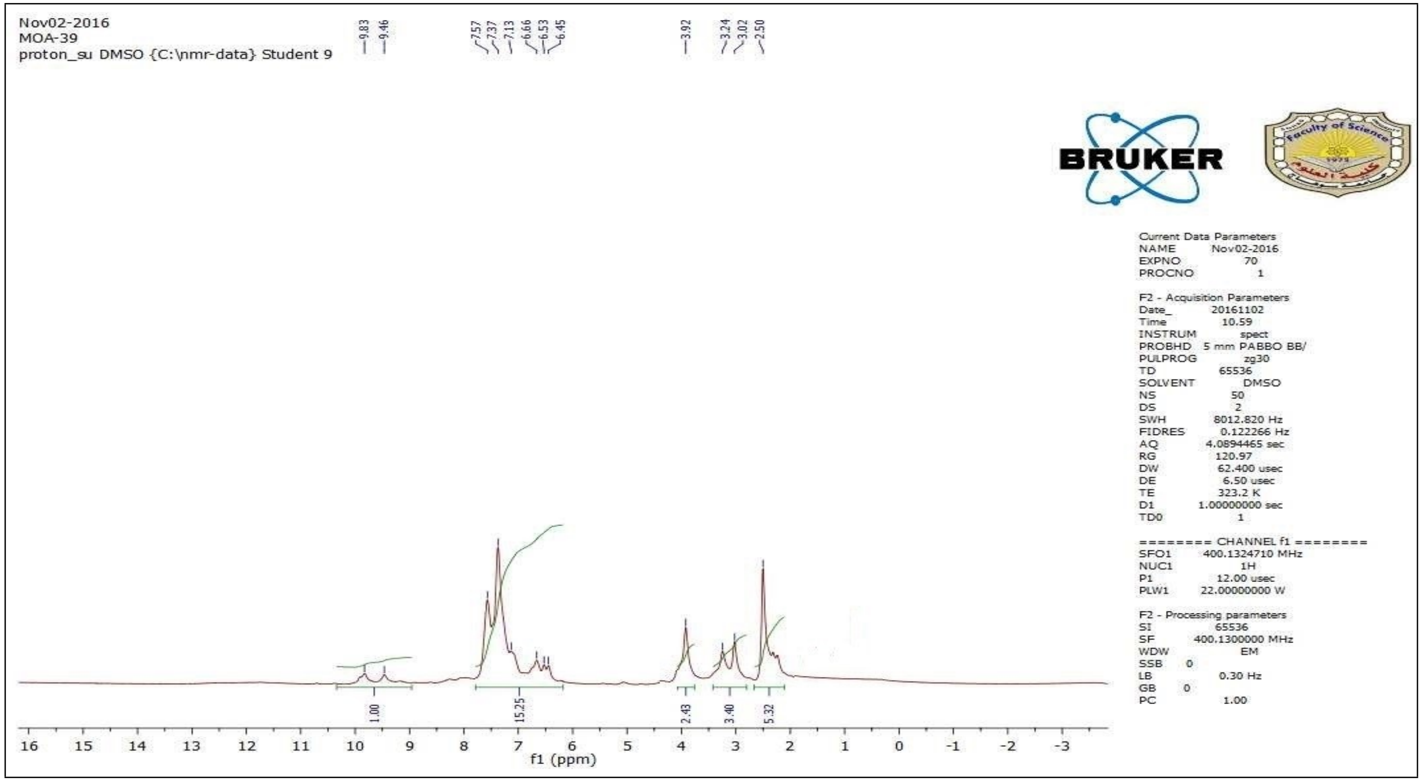


**Figure S5.** $^{1}$H NMR Spectrum of Compound (3)

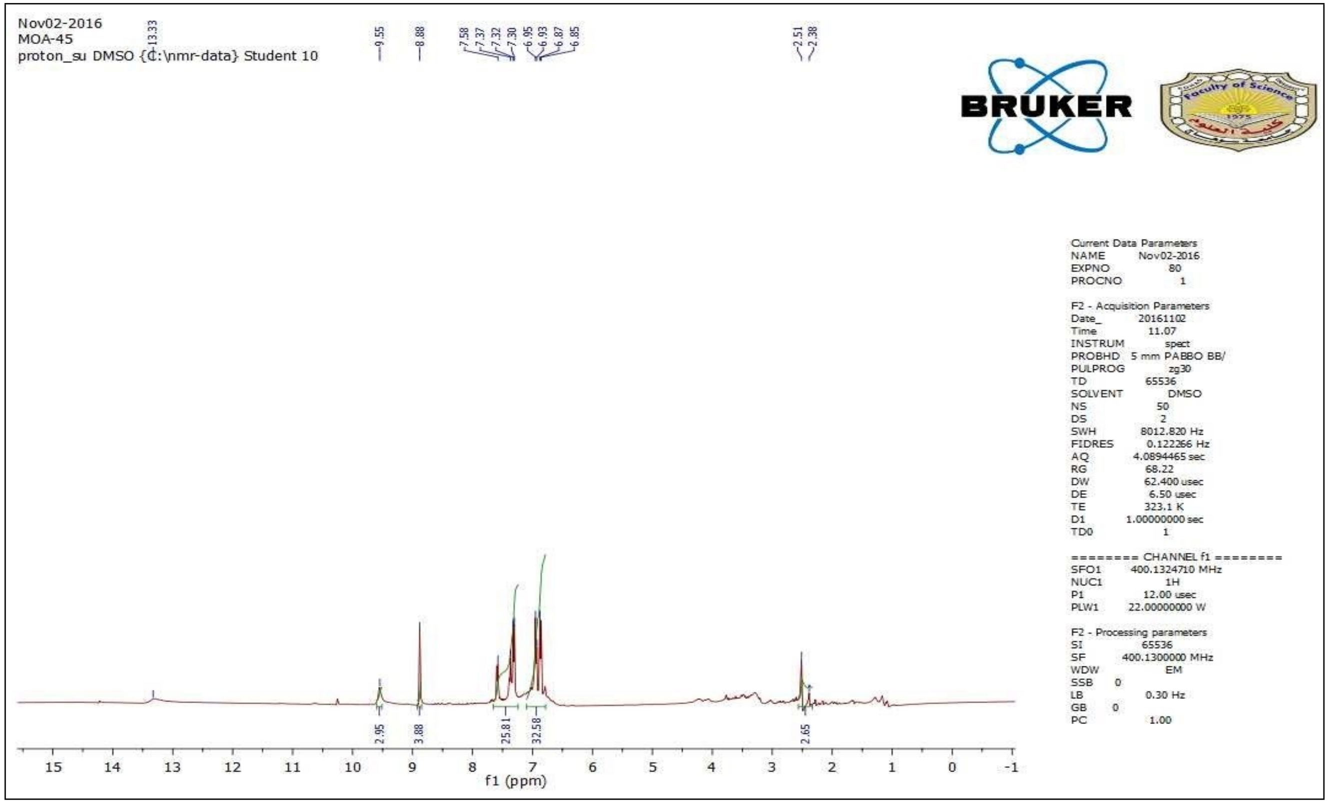


**Figure S6.** $^{1}H$ NMR Spectrum of Compound (4)

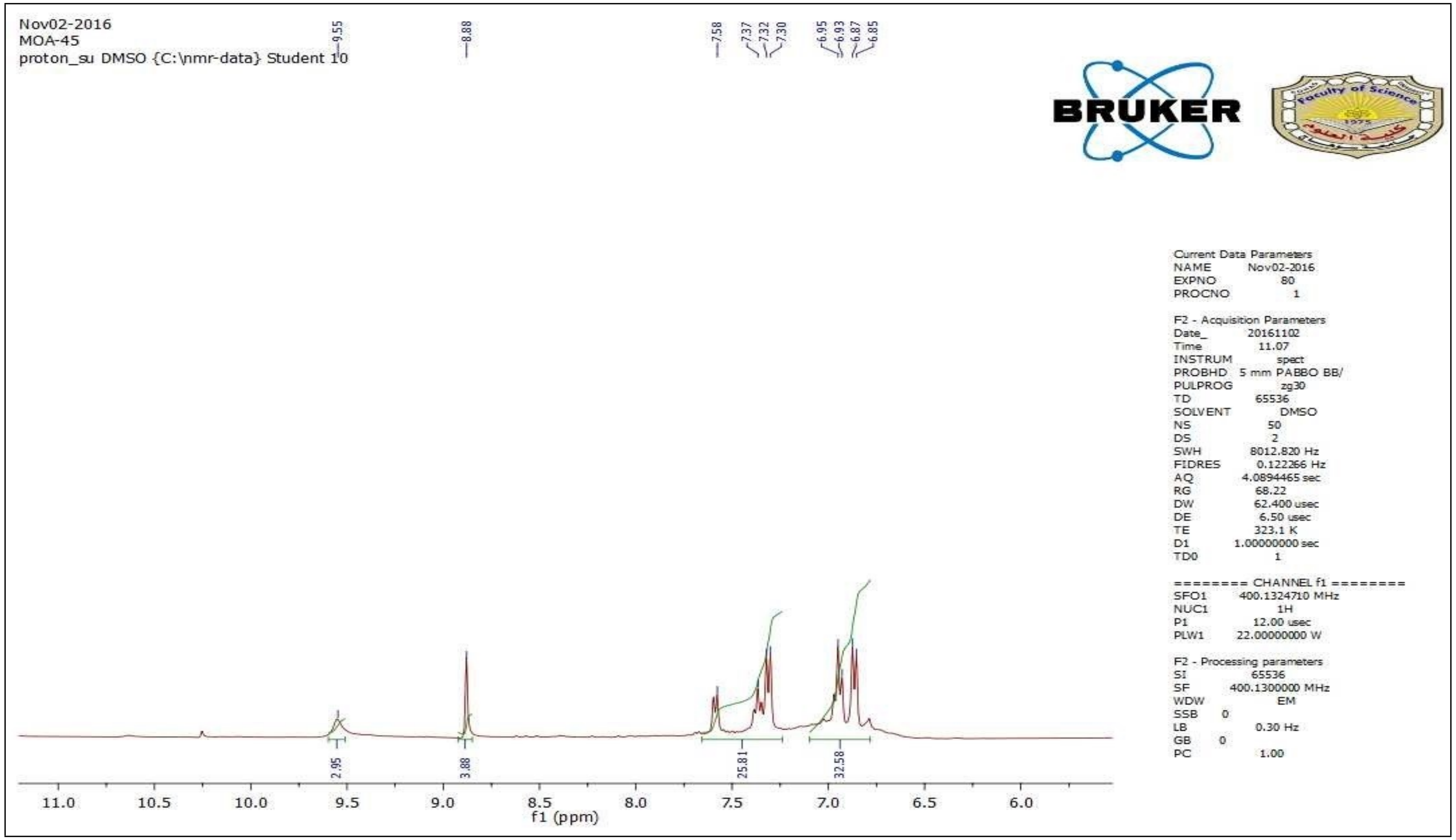


**Figure S7.** $^{1}$H NMR Spectrum of Compound (4)

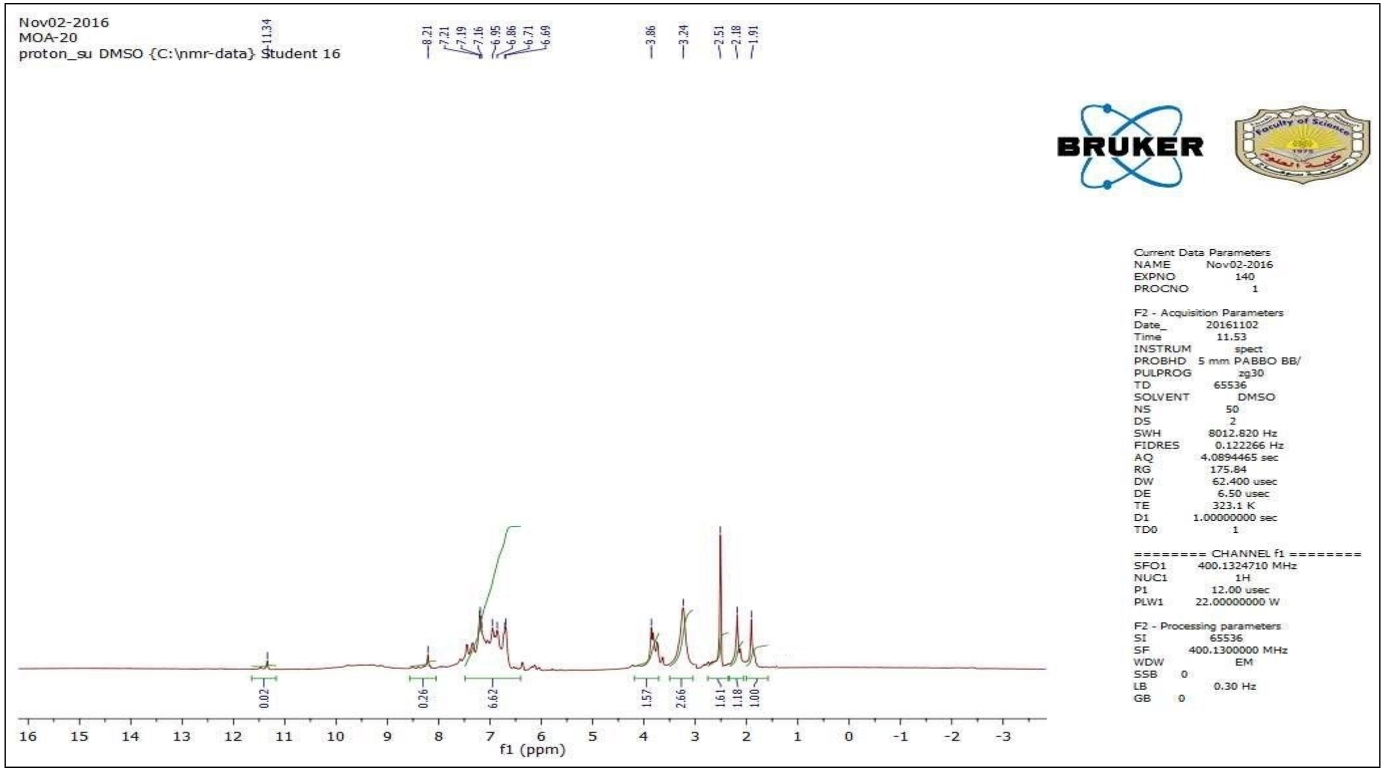


**Figure S8.** $^{1}H$ NMR Spectrum of Compound (5)

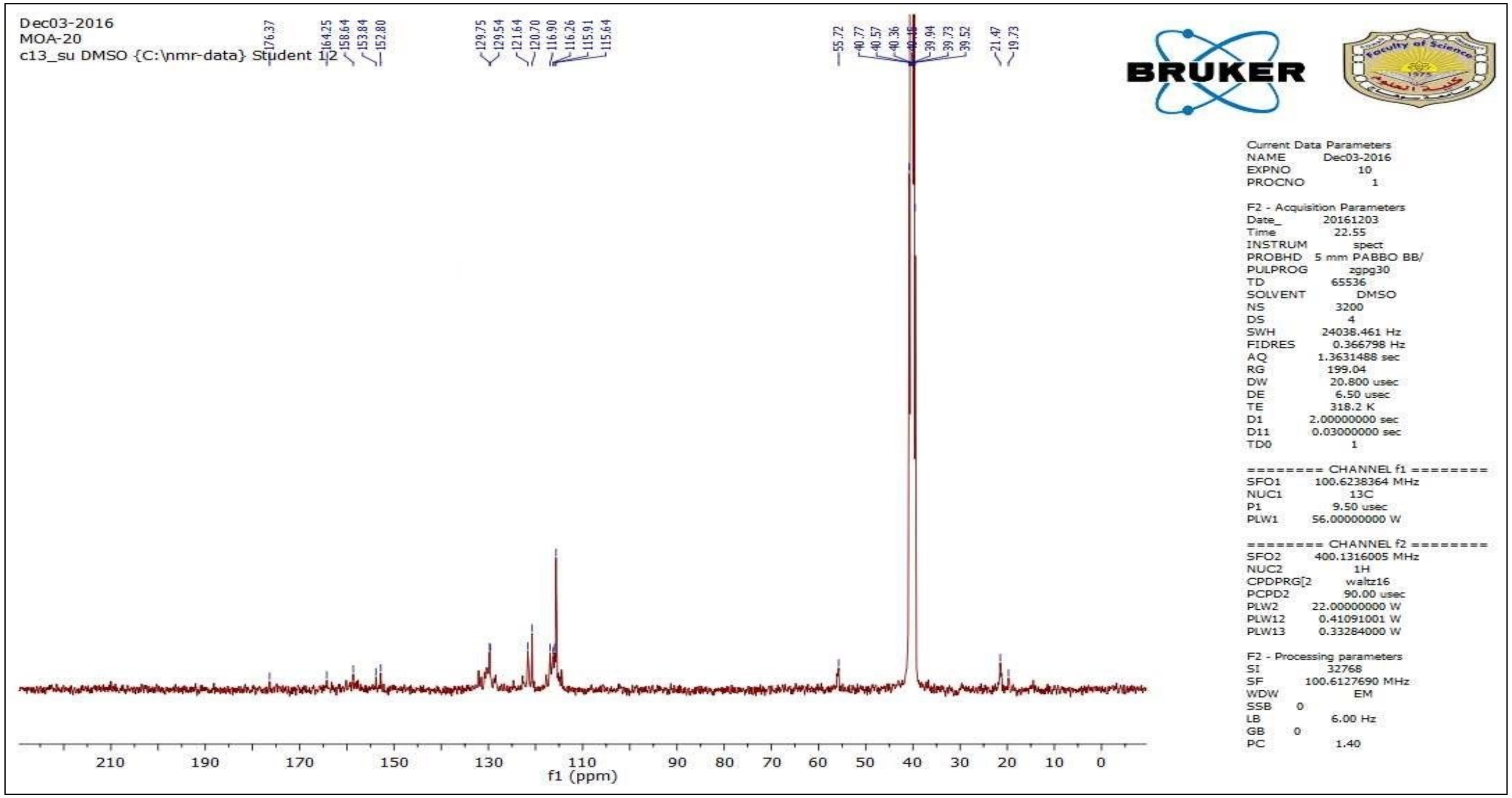


**Figure S9.** $^{13}$C NMR Spectrum of Compound (5)

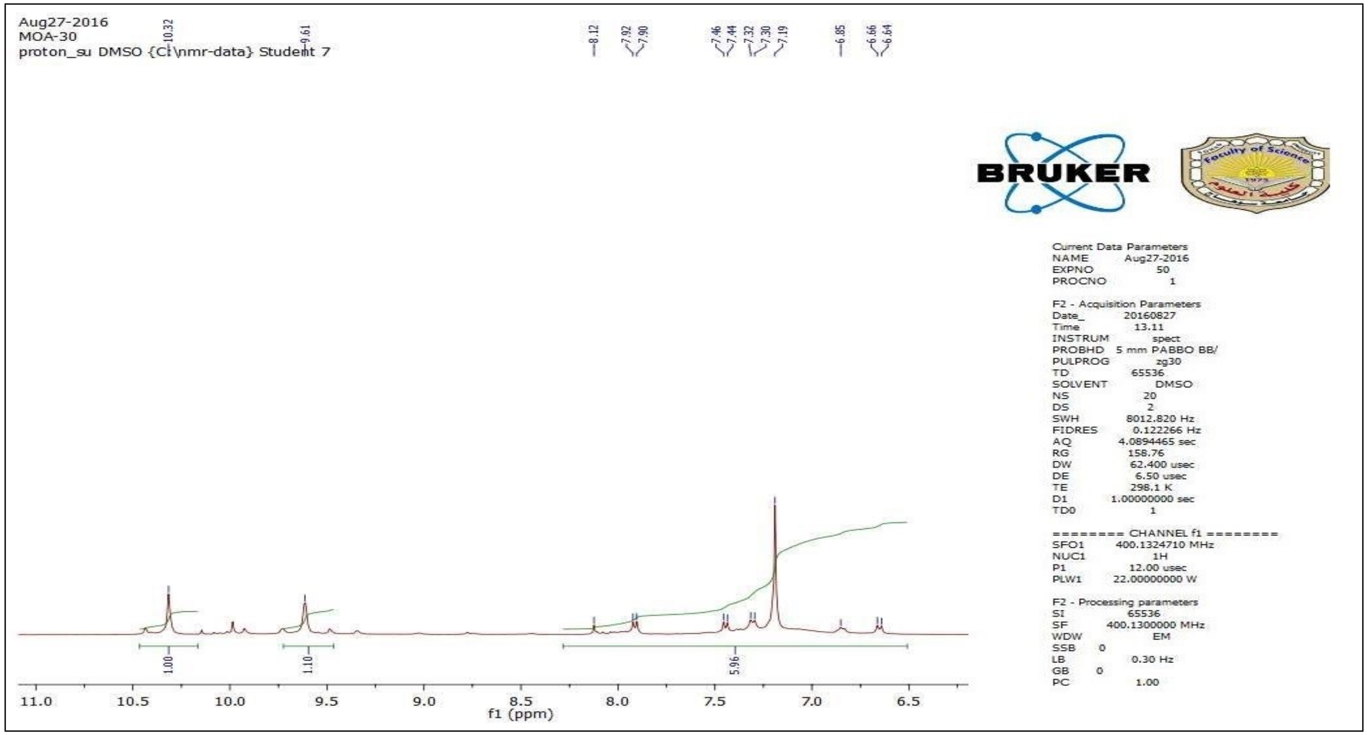


**Figure S10.** $^1$H NMR Spectrum of Compound (6a)

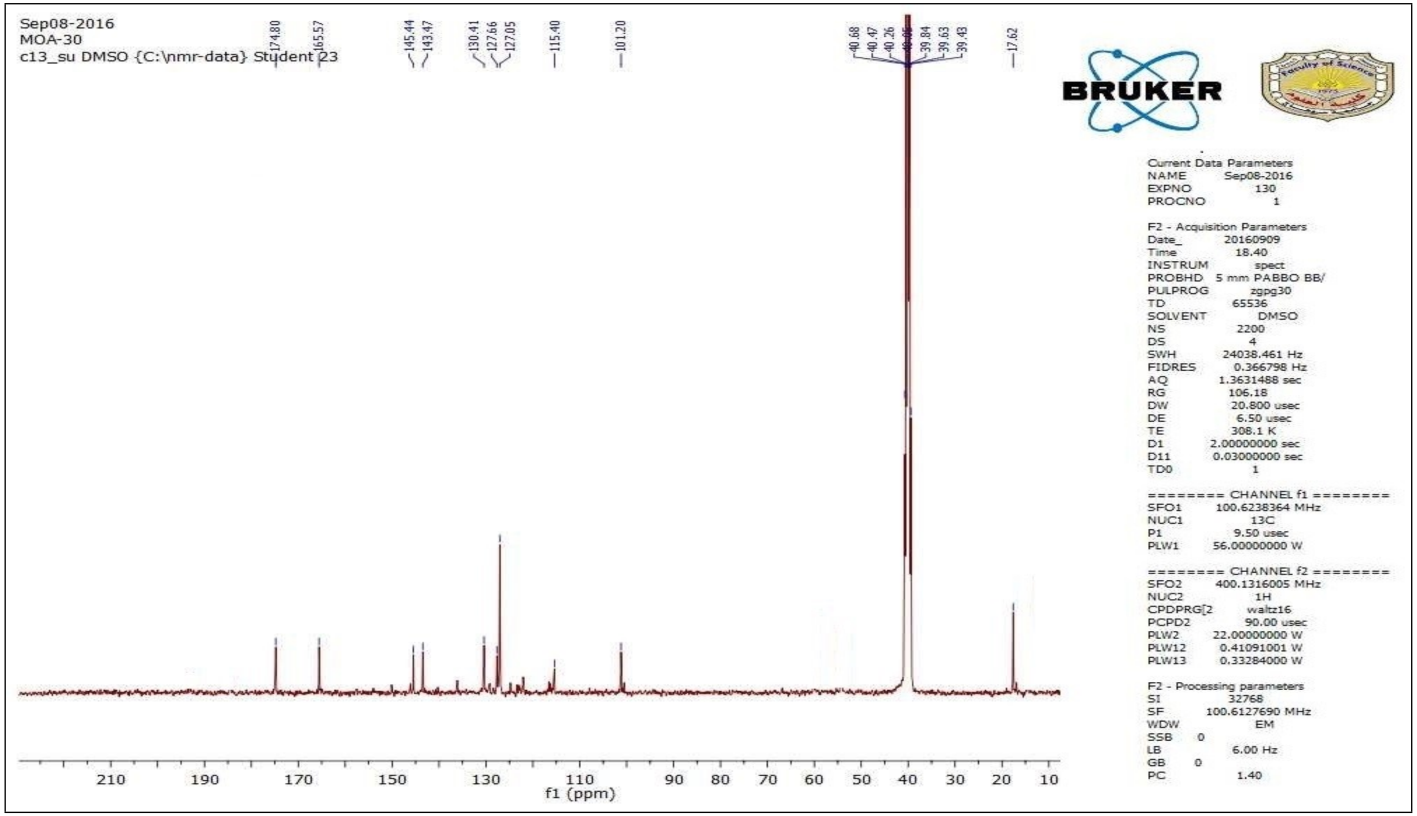


**Figure S11.** $^{13}$C NMR Spectrum of Compound (6a)

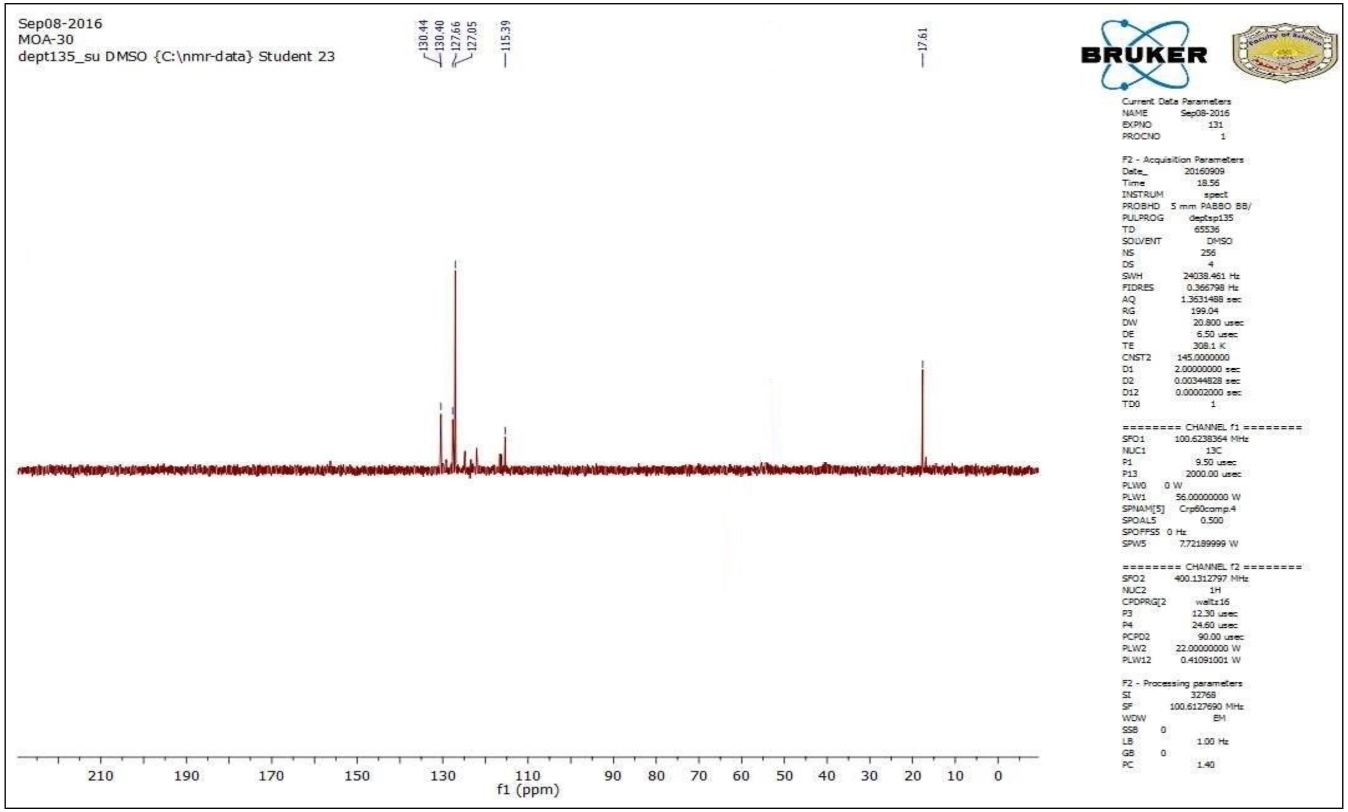


**Figure S12.** DEPT 135 $^{13}$C NMR Spectrum of Compound (6a)

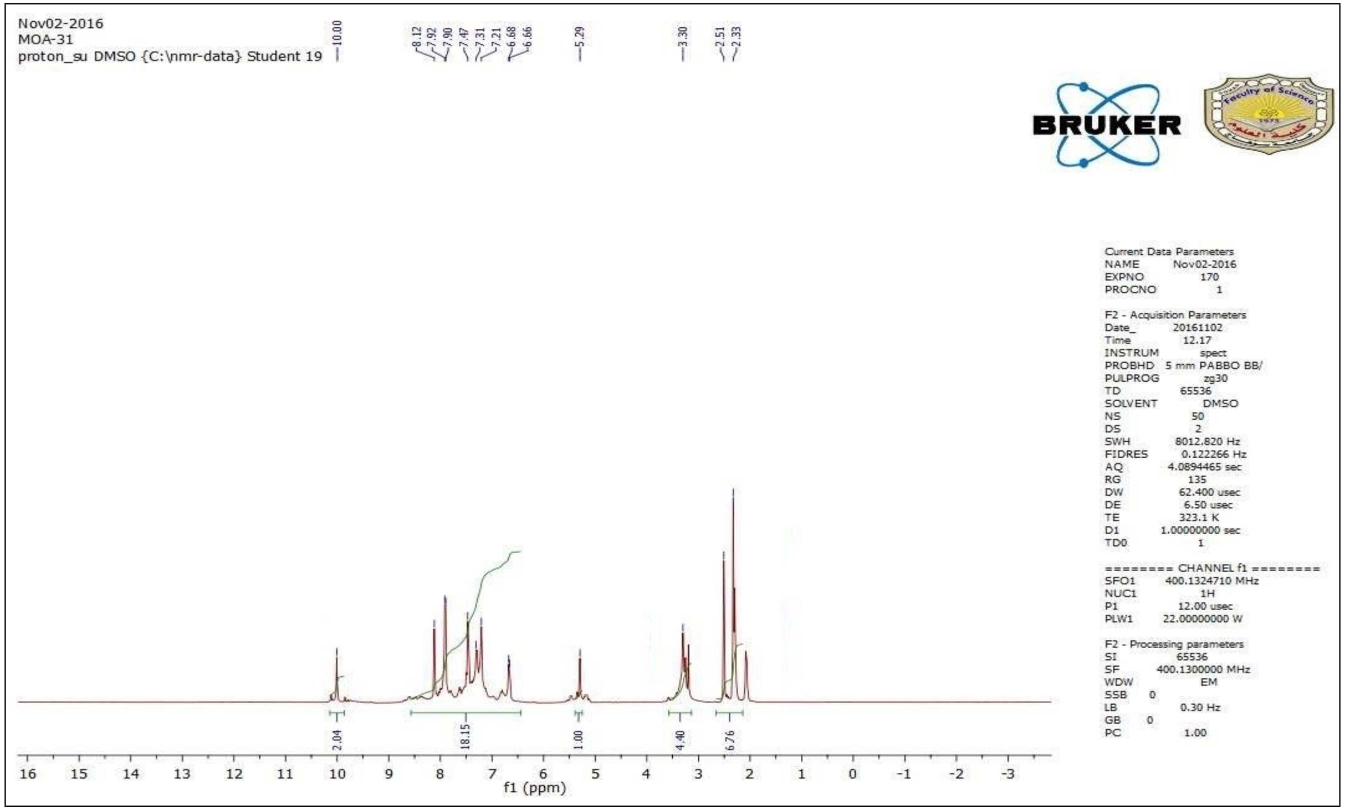


**Figure S13.** $^{1}$H NMR Spectrum of Compound (6b)

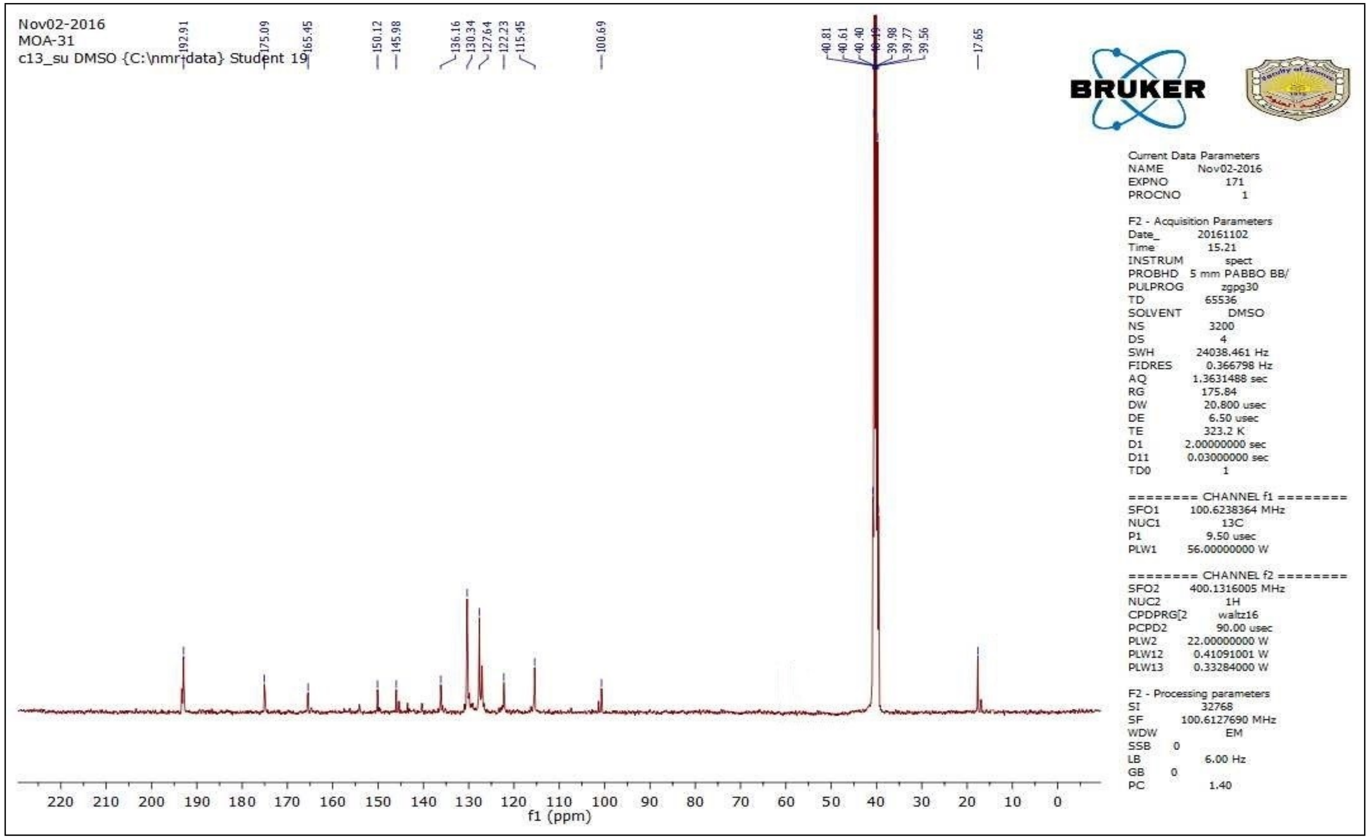


**Figure S14.** $^{13}$C NMR Spectrum of Compound (6b)

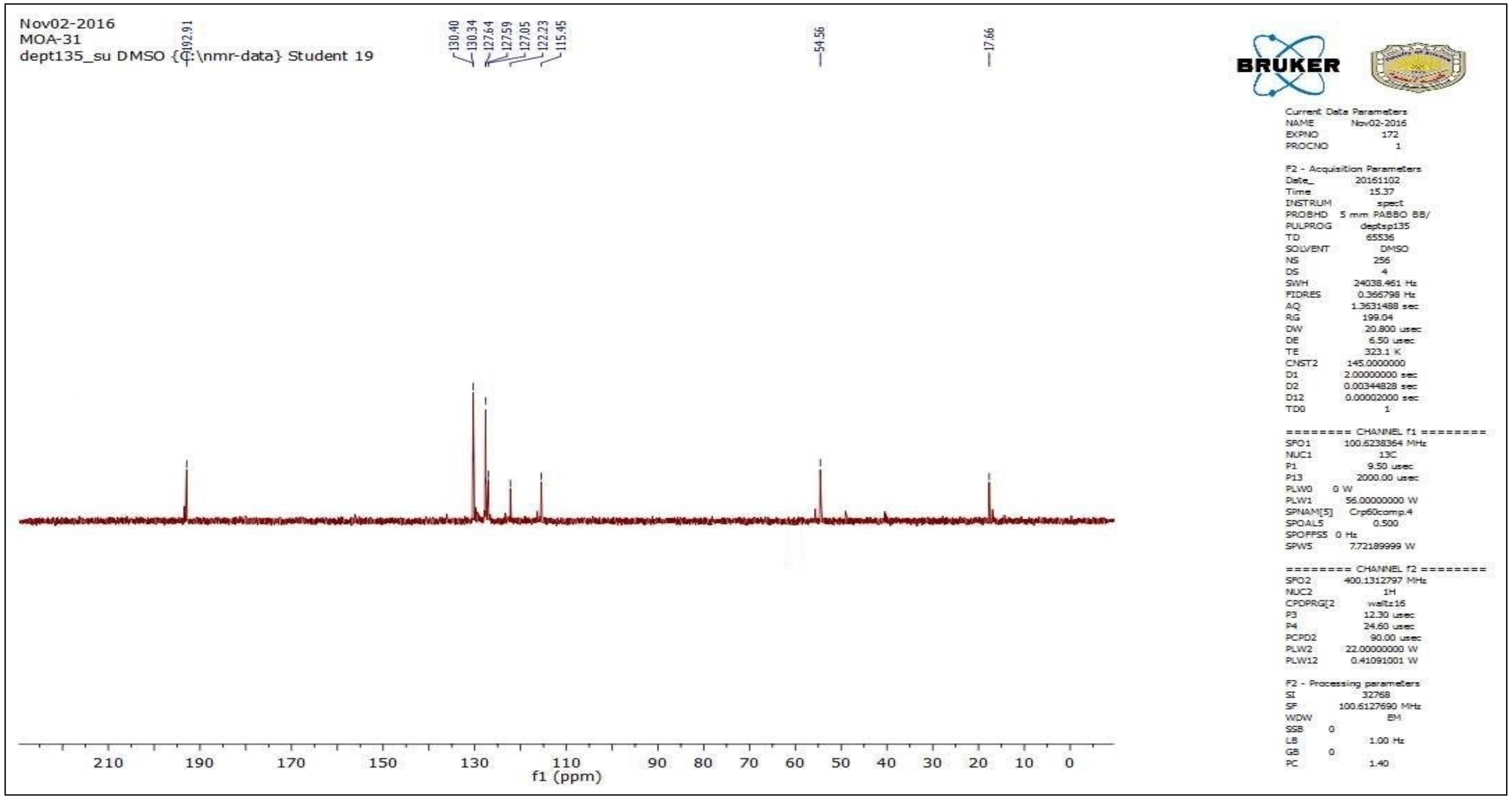


**Figure S15.** DEPT 135 $^{13}C$ NMR Spectrum of Compound (6b)

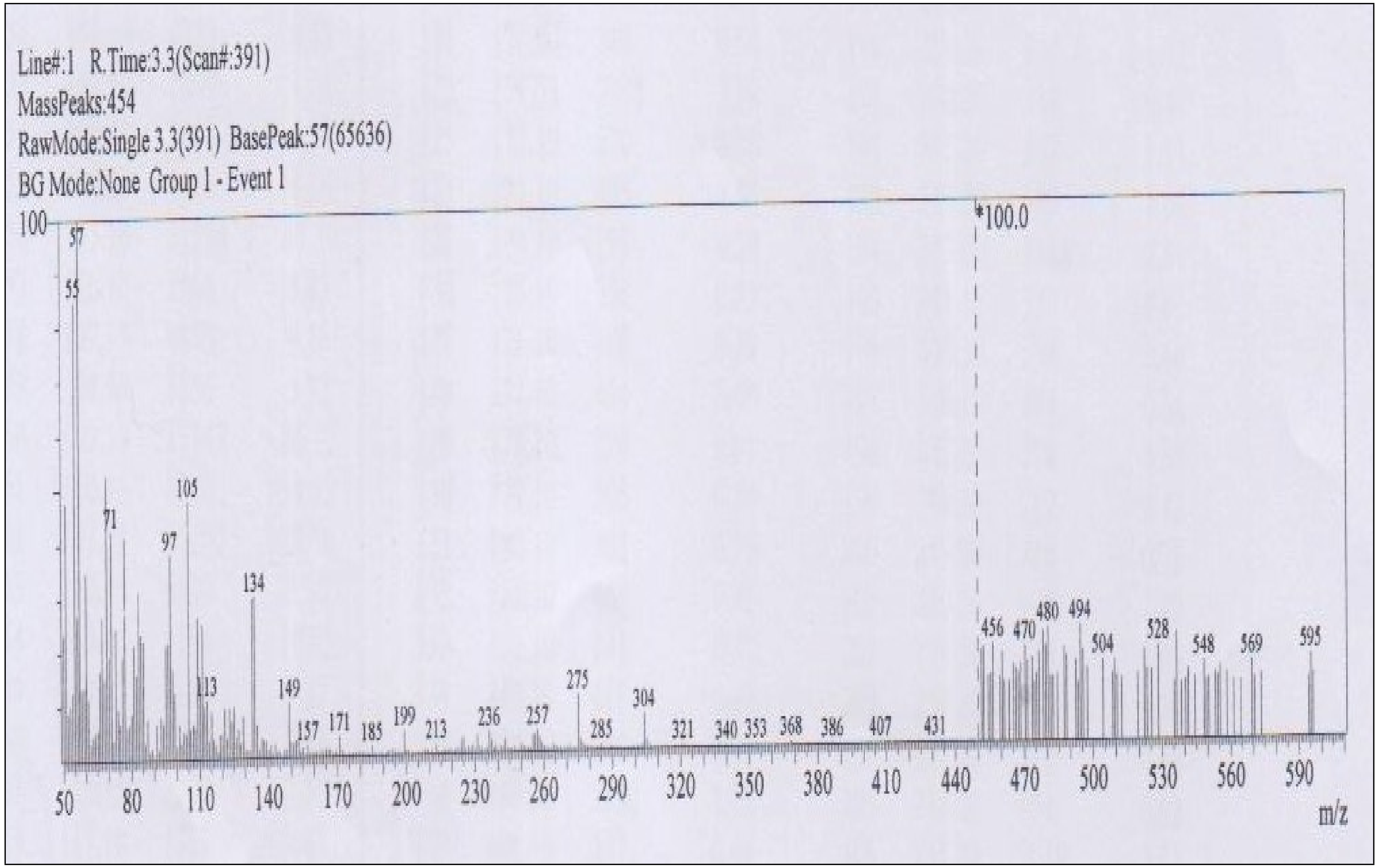


**Figure S16.** Mass Spectrum of Compound (6b)

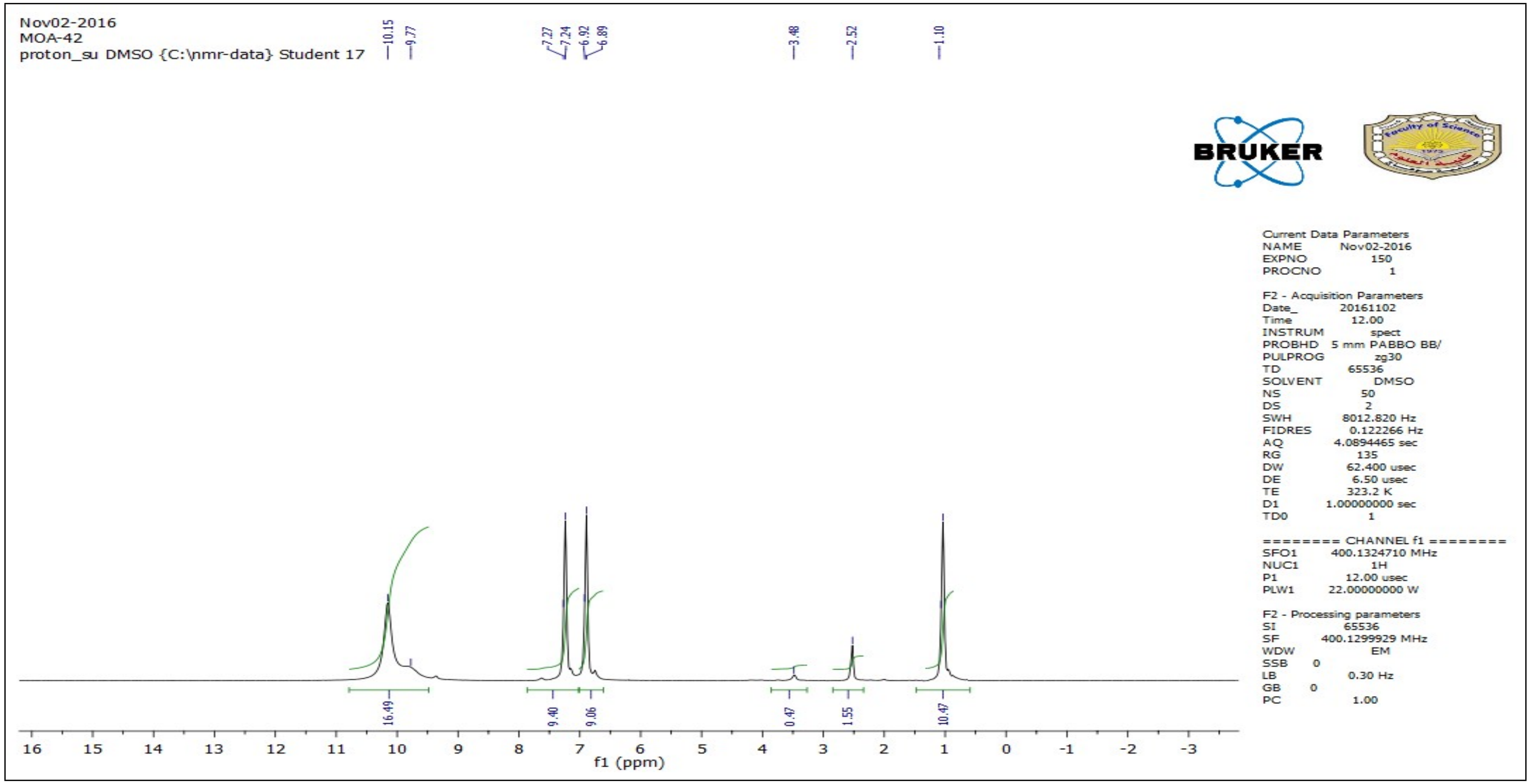


**Figure S17.** $^{1}$H NMR Spectrum of Compound (7)

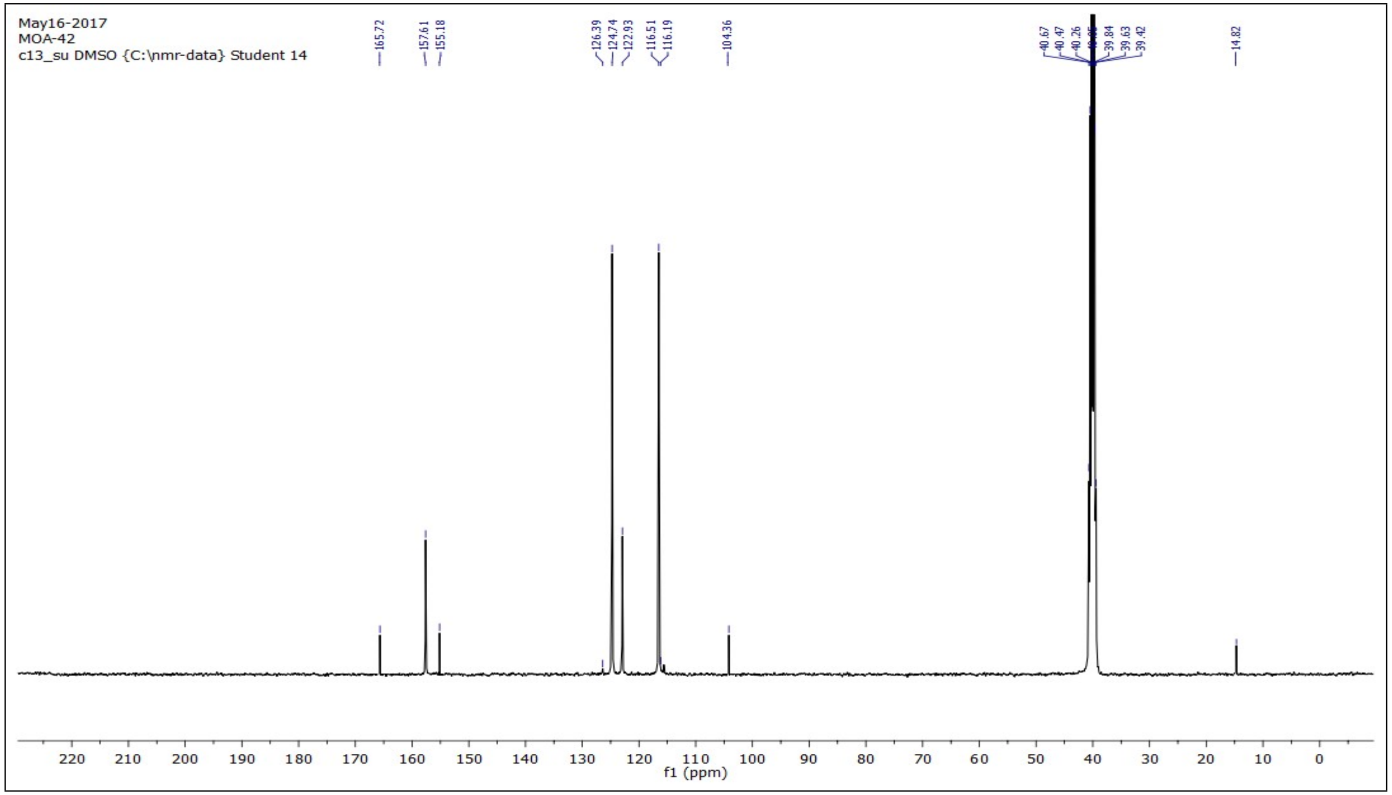


**Figure S18.** $^{13}$C NMR Spectrum of Compound (7)

23-May-17 17:21:42

**Cairo University**
**Micro Analytical Center**

***DI Analysis***
***Shimadzu Qp-2010 Plus***

Sample Information

| | |
|---|---|
| Analyzed by | : Noha Bagato. |
| Analyzed | : 23/05/2017 05:13:03 م |
| Sample Name | : MOA 42 |
| Sample ID | : |
| Customer Name | : علوم القاهرة - د. جهاد جنيدي - نسمة |
| Data File | : C:\GCMSsolution\Data\Project1\MOA 42.QGD |
| Org Data File | : C:\GCMSsolution\Data\Project1\MOA 42.QGD |
| Method File | : C:\GCMSsolution\Data\Project1\High Temperature Op |
| Org Method File | : C:\GCMSsolution\Data\Project1\High Temperature Op |
| Report File | : |
| Tuning File | : C:\GCMSsolution\System\Tune1_default.qgt |
| $EndIf$Modified by | : Noha Bagato. |
| Modified | : 23/05/2017 05:19:08 م |

Method

===== Analytical Line 1 =====

| | |
|---|---|
| IonSourceTemp | :250.00 °C |
| [MS Table] | |
| --Group 1 - Event 1-- | |
| Start Time | :0.00min |
| End Time | :10.00min |
| ACQ Mode | :Scan |
| Event Time | :0.50sec |
| Scan Speed | :1428 |
| Start m/z | :50.00 |
| End m/z | :700.00 |
| Electron Voltage | : 70 eV |
| Ionization Mode | : EI |

C:\GCMSsolution\Data\Project1\MOA 42.QGD

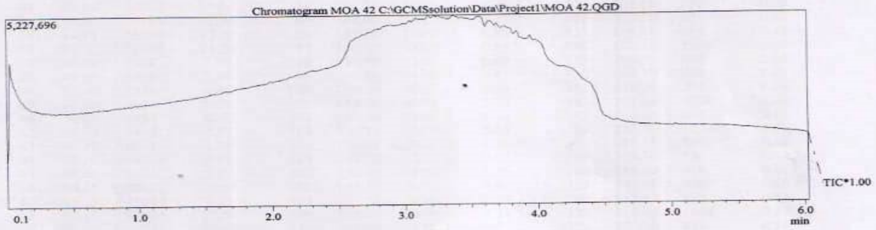


Spectrum

Line#:1 R.Time:5.4(Scan#:649)
RawMode:Single 5.4(649) BasePeak:109(151566)
BG Mode:None Group 1 - Event 1

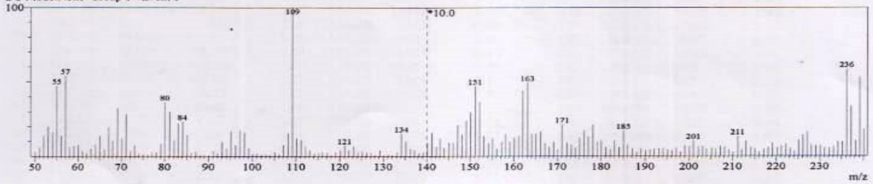


Mass Table
Line#:1 R.Time:5.4(Scan#:649)
MassPeaks:191
RawMode:Single 5.4(649) BasePeak:109(151566)
BG Mode:None Group 1 - Event 1

| # | m/z | Abs. In | Rel. Int. | # | m/z | Abs. In | Rel. Int. | # | m/z | Abs. In | Rel. Int. |
|---|---|---|---|---|---|---|---|---|---|---|---|
| 1 | 50.00 | 4220 | 2.78 | 4 | 53.05 | 30180 | 19.91 | 7 | 56.05 | 21125 | 13.94 |
| 2 | 51.00 | 9295 | 6.13 | 5 | 54.05 | 23974 | 15.82 | 8 | 57.05 | 81868 | 54.01 |
| 3 | 52.05 | 20728 | 13.68 | 6 | 55.05 | 71625 | 47.26 | 9 | 58.05 | 9073 | 5.99 |

1 / 2

**Figure S19.** Mass Spectrum of Compound (7)

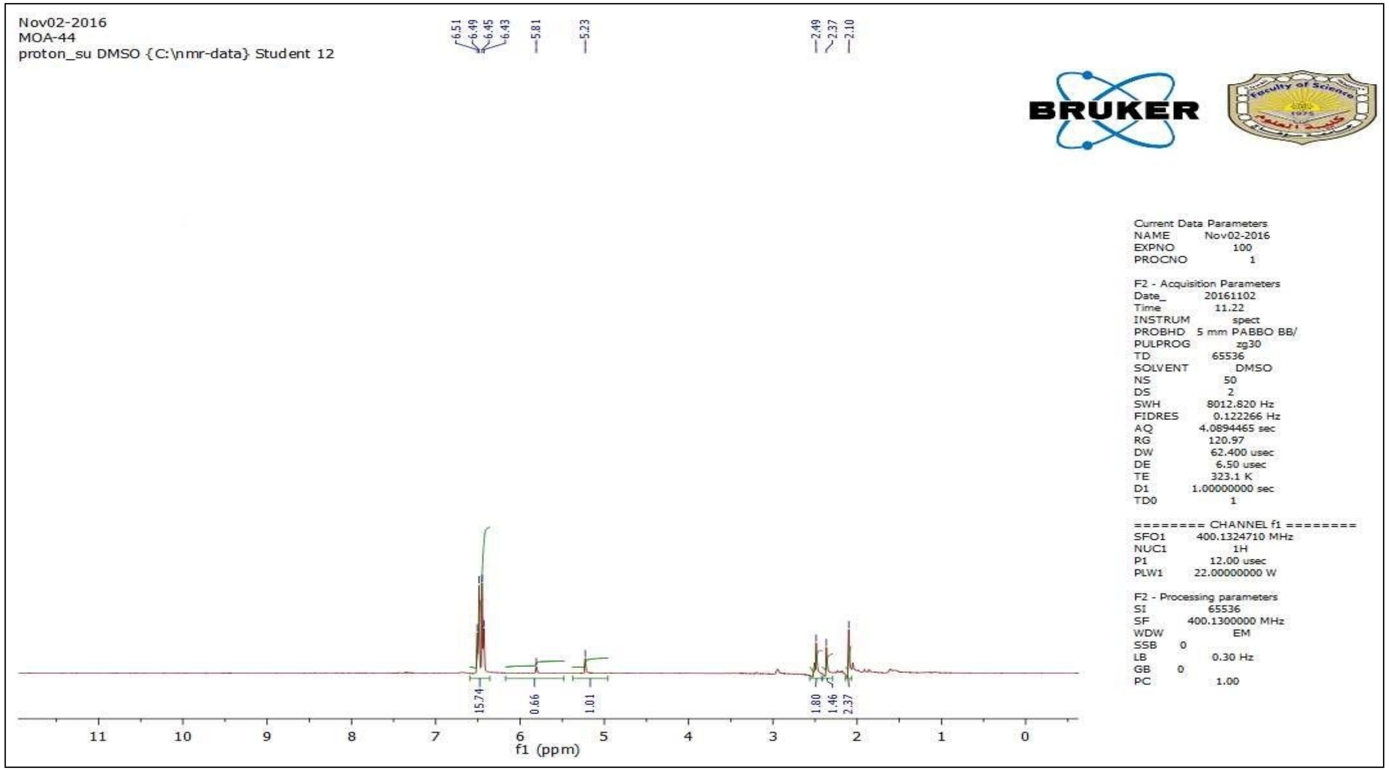


**Figure S20.** $^{1}$H NMR Spectrum of Compound (8)

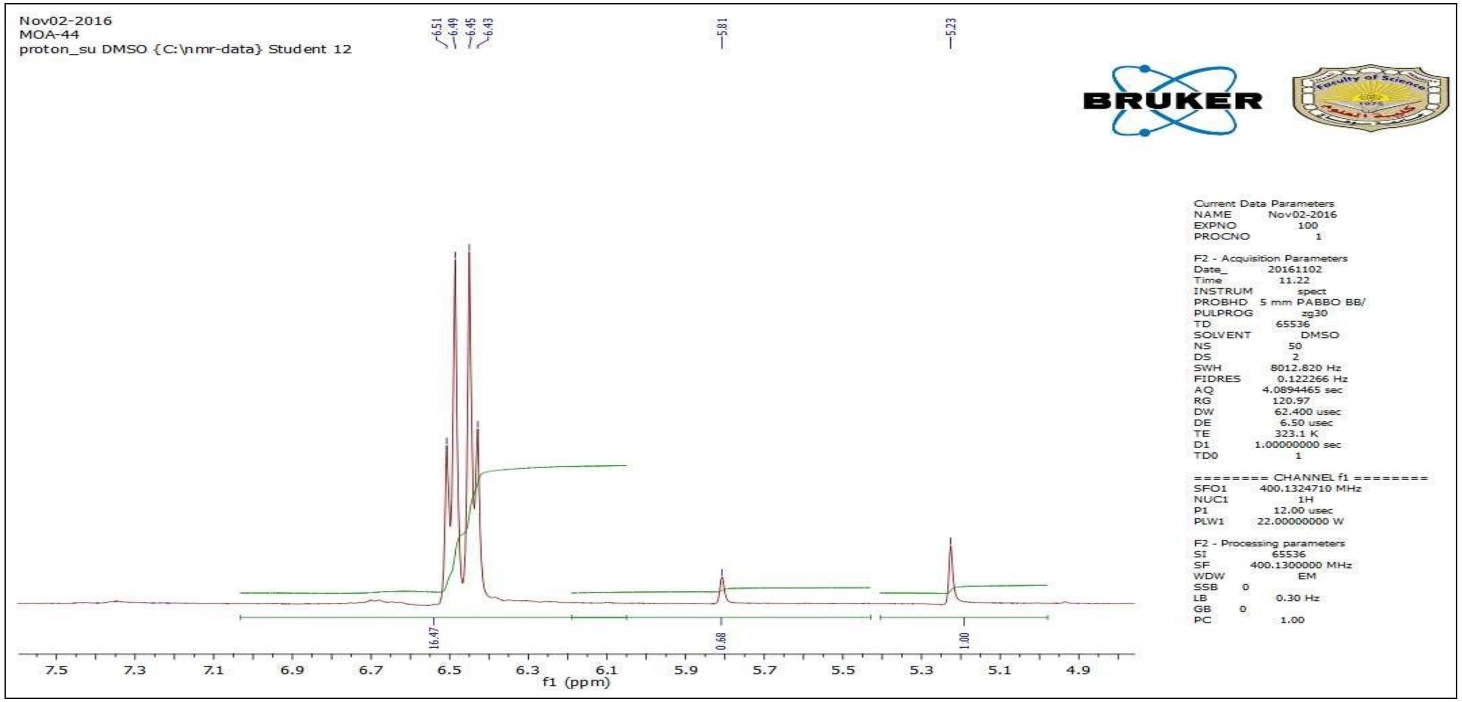


**Figure S21.** $^{1}H$ NMR Spectrum of Compound (8)

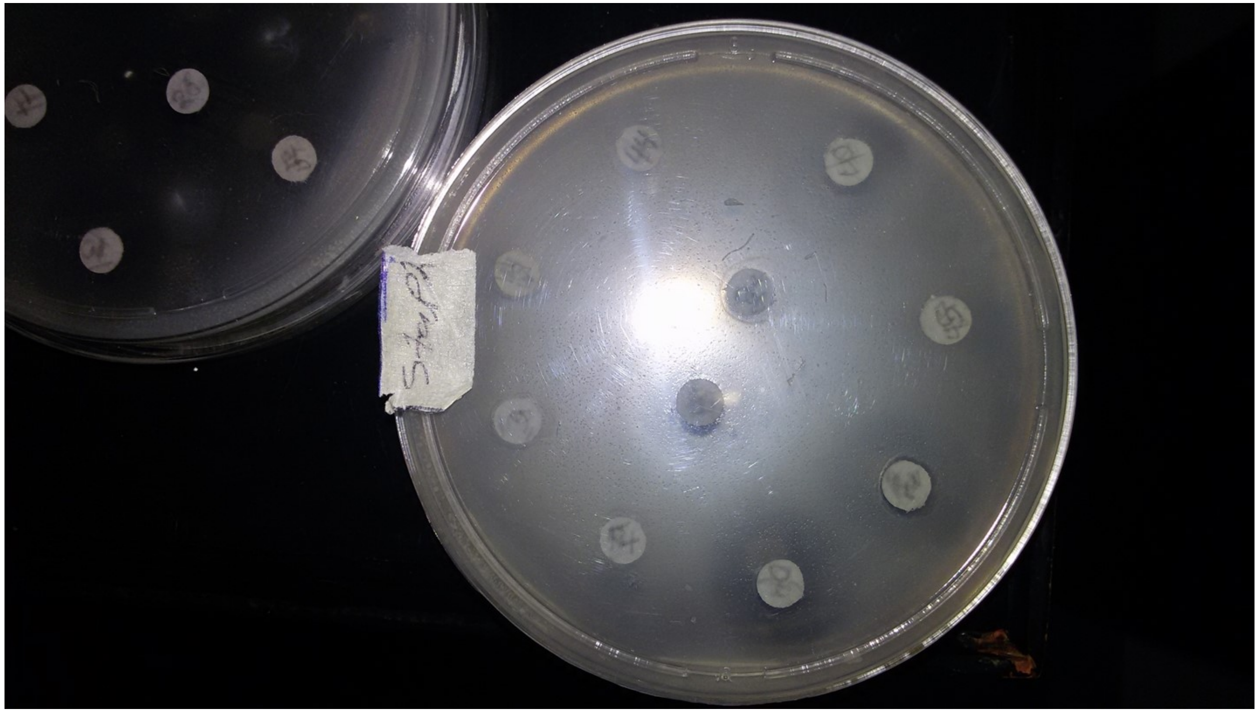
Staph

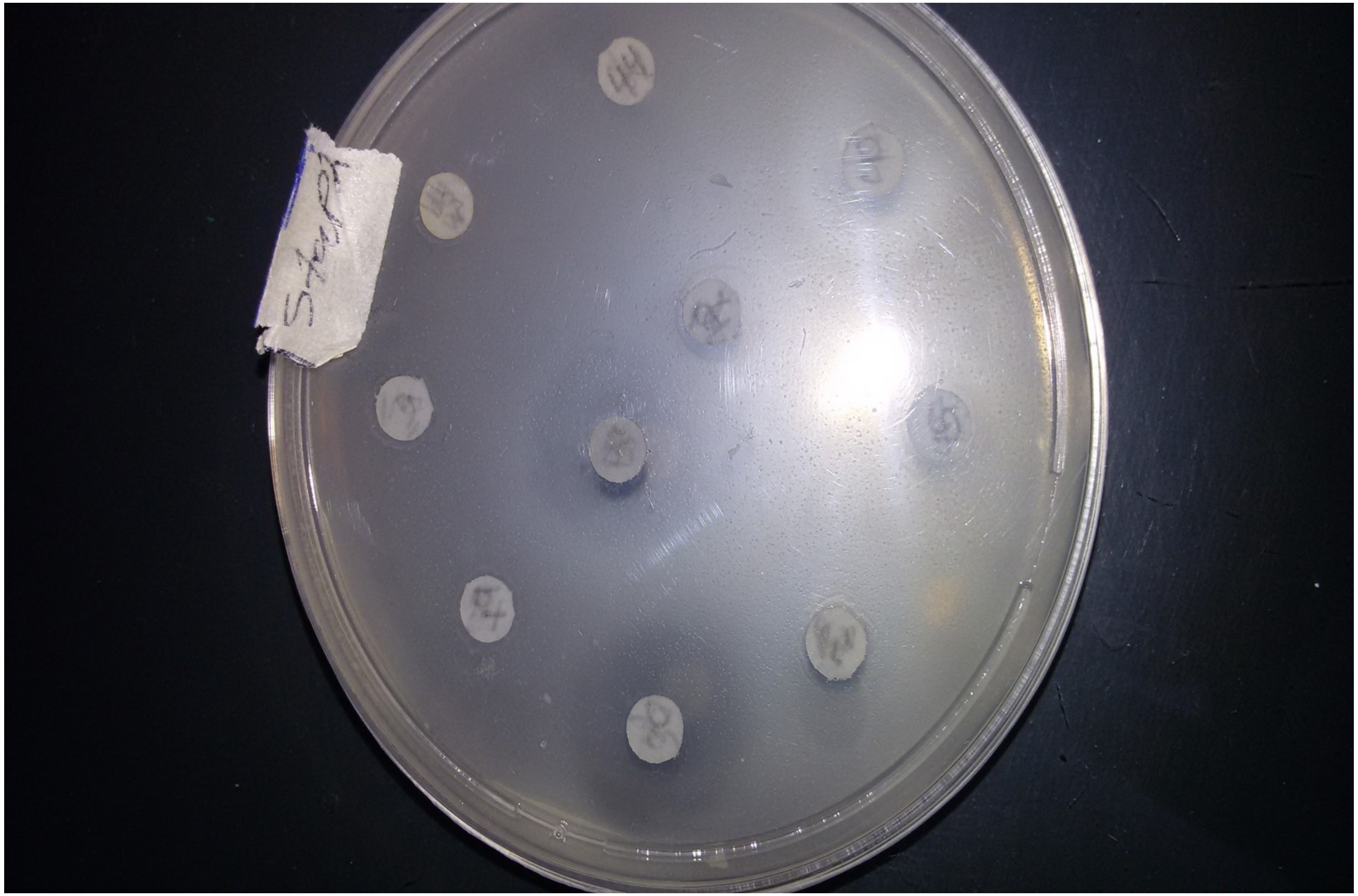

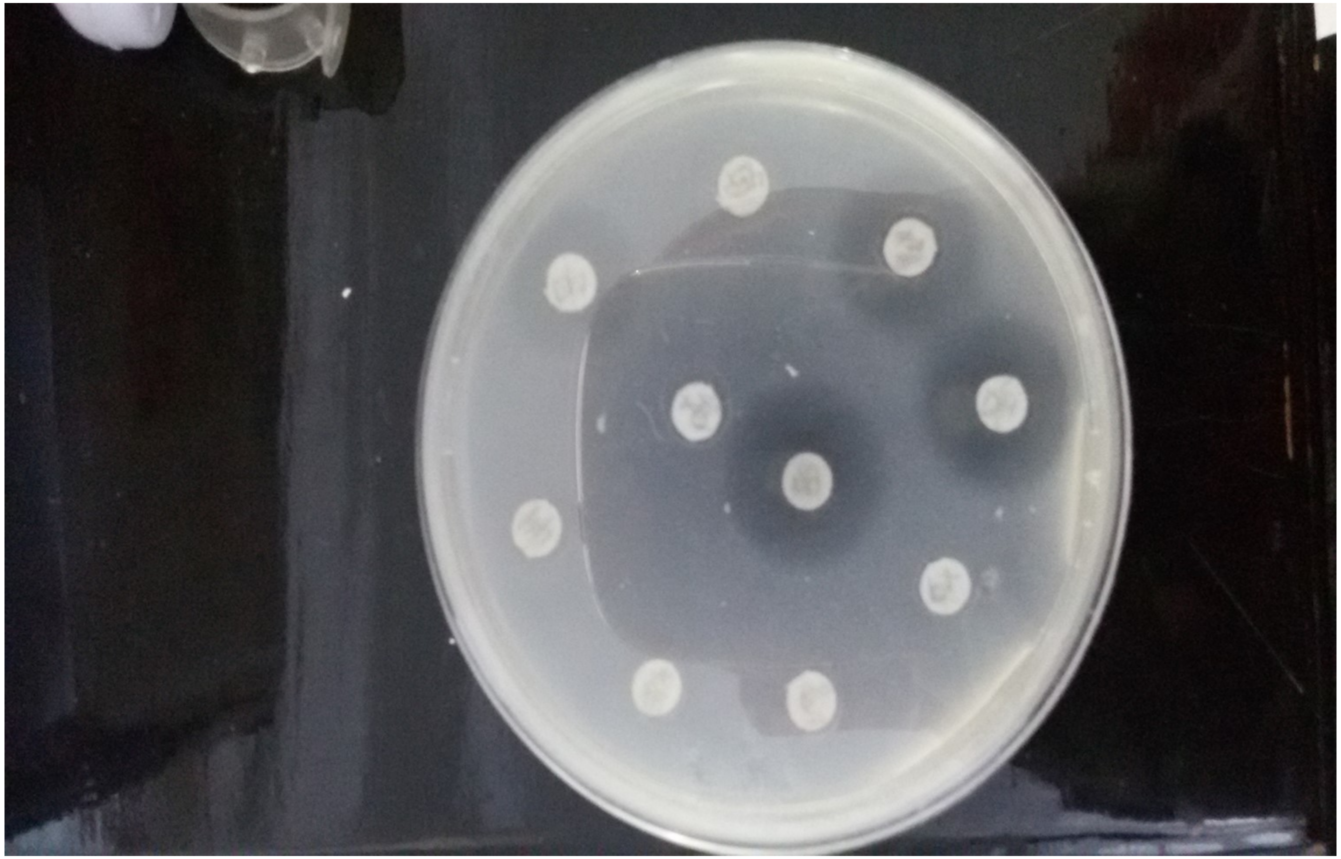

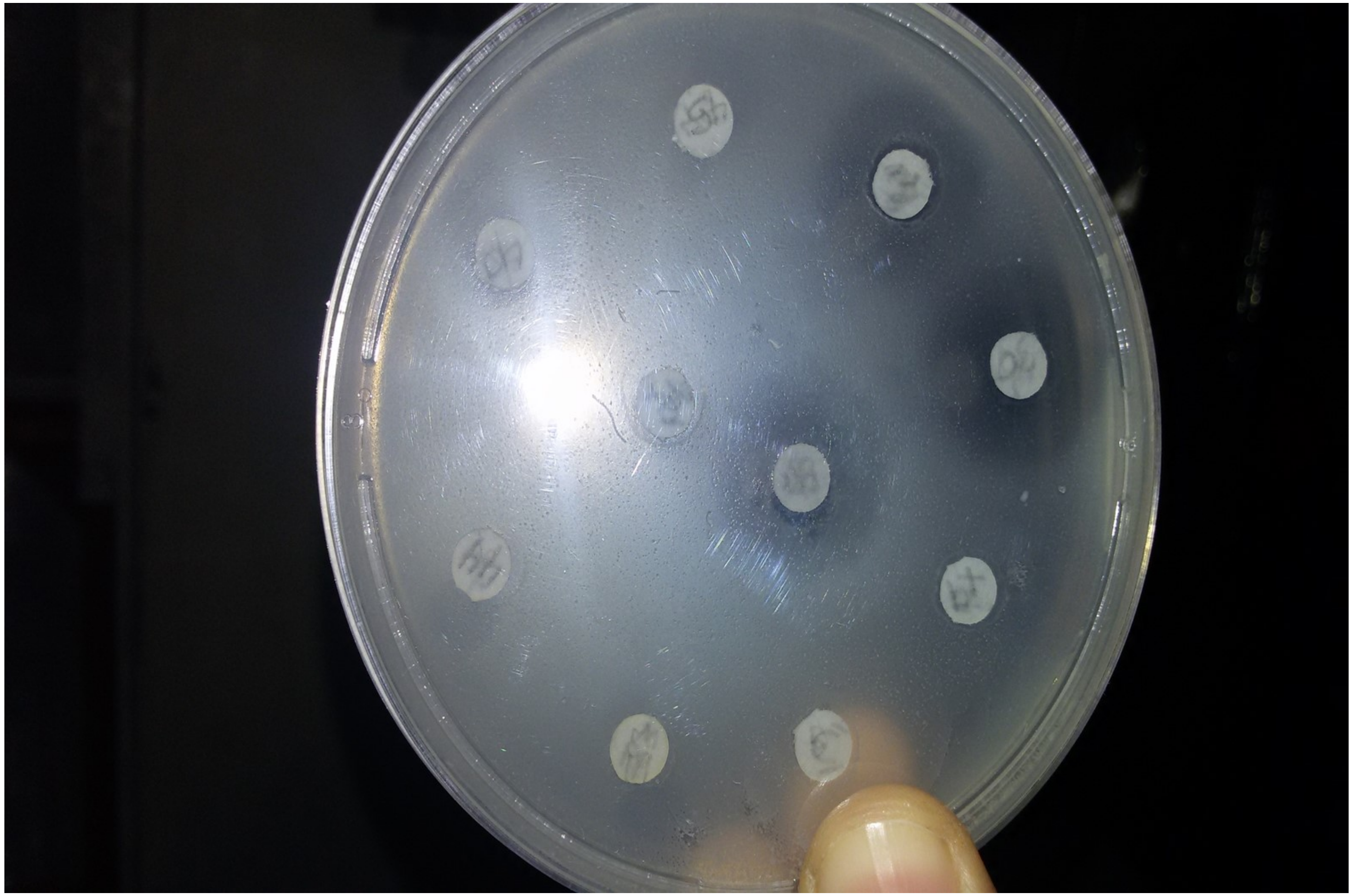

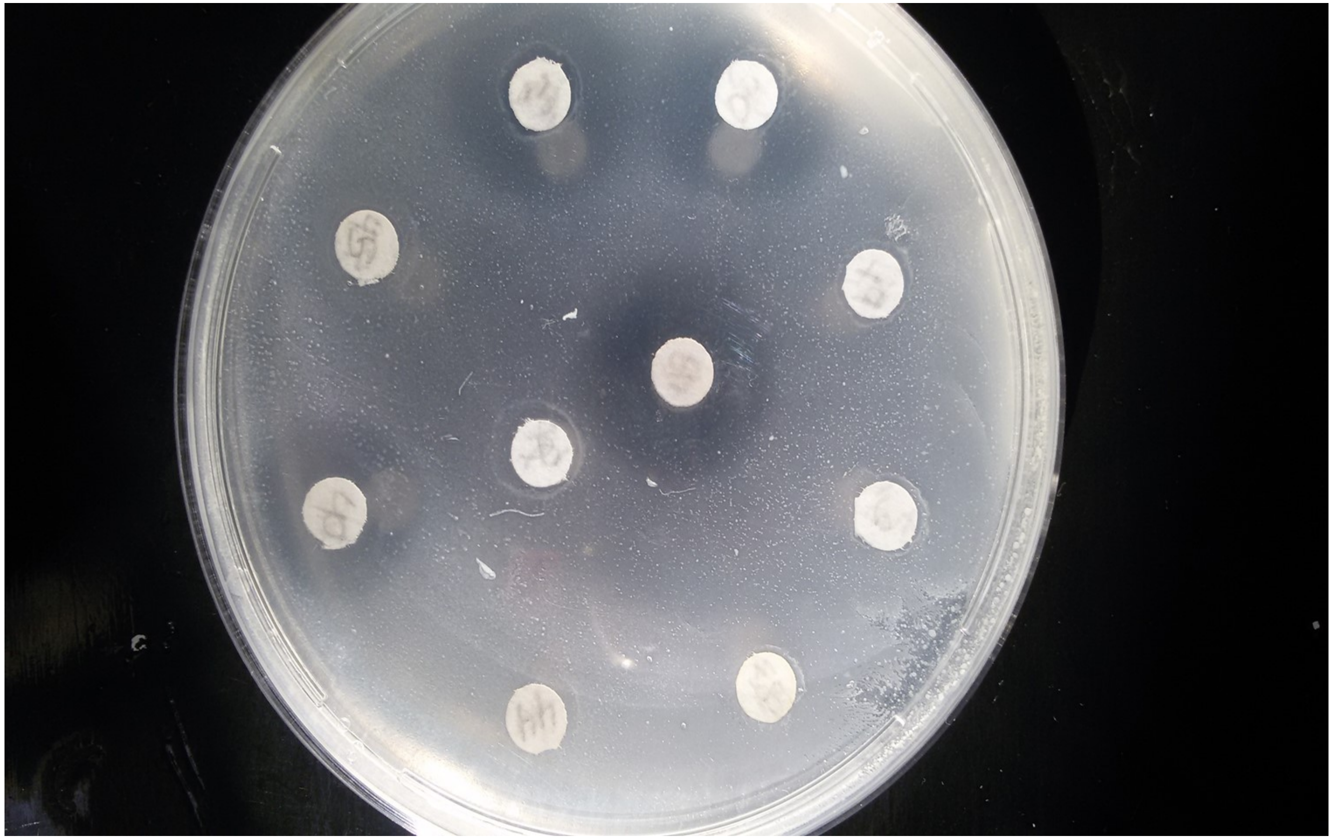

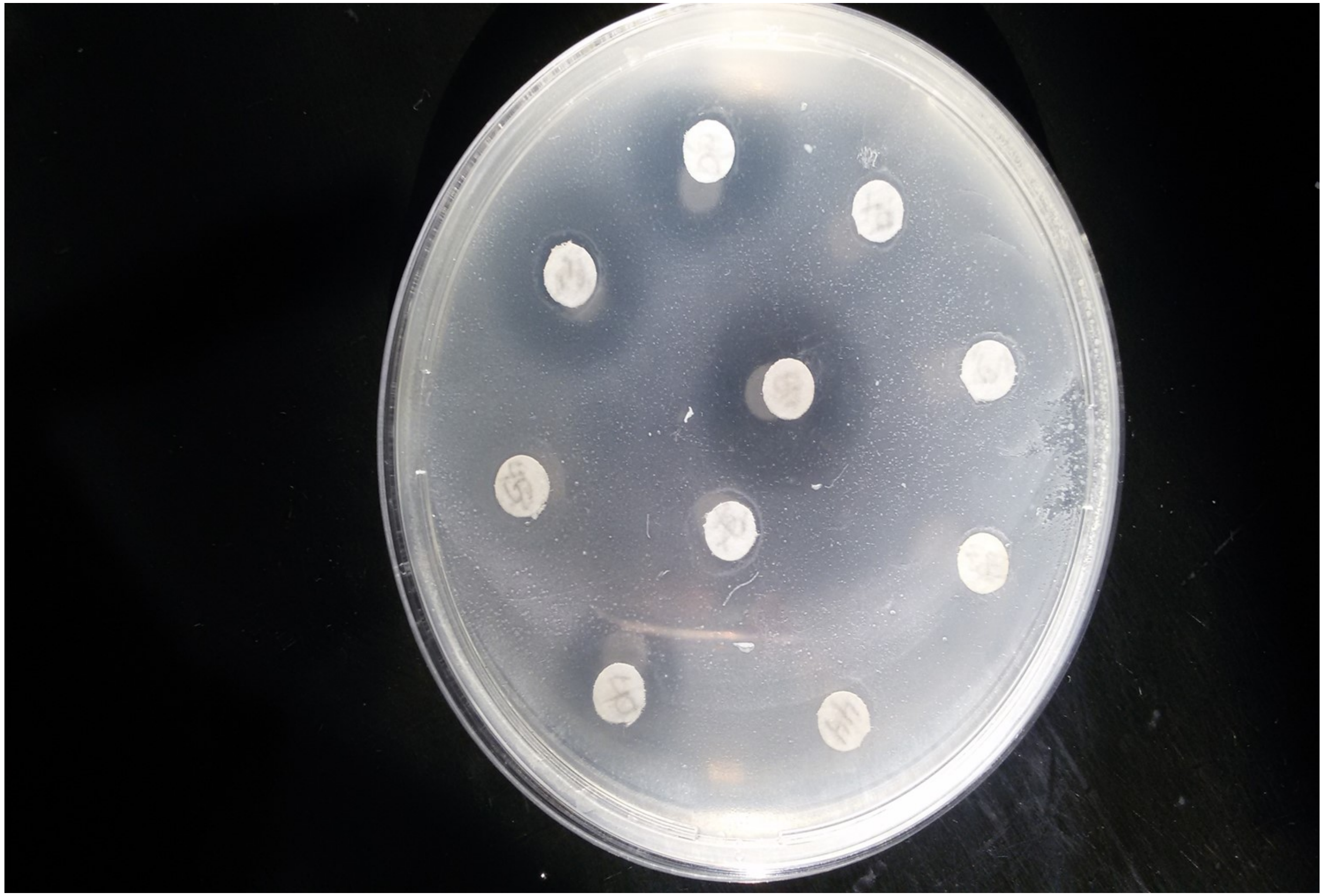

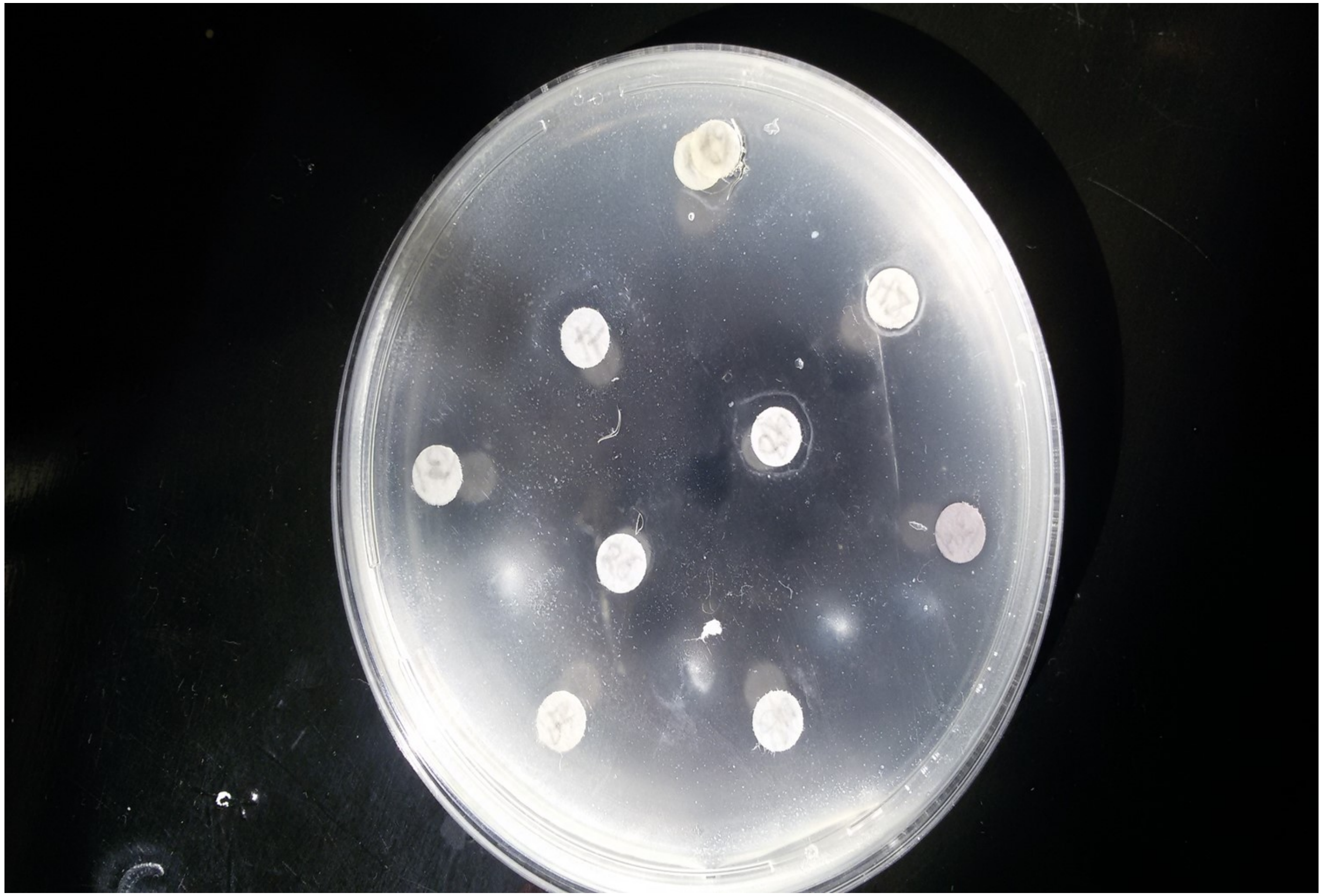

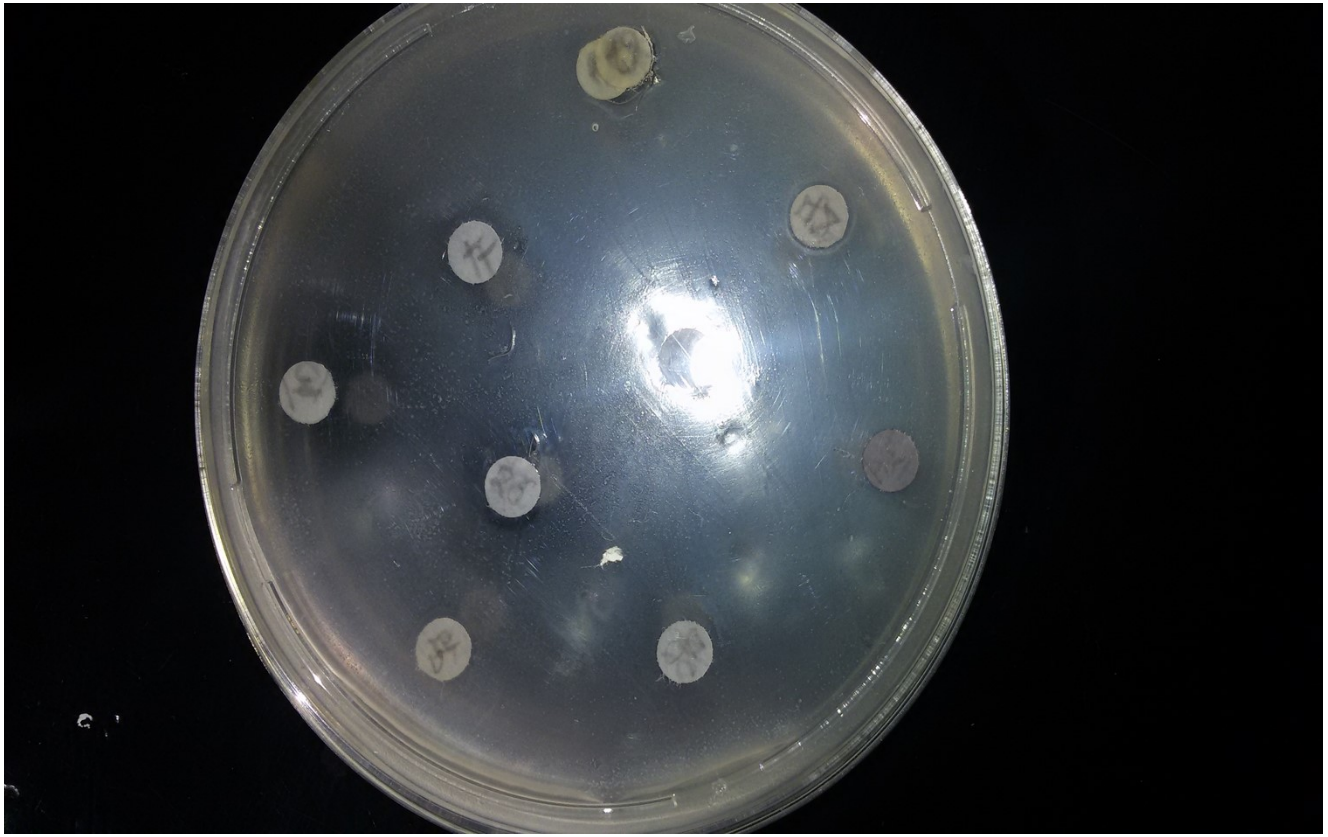

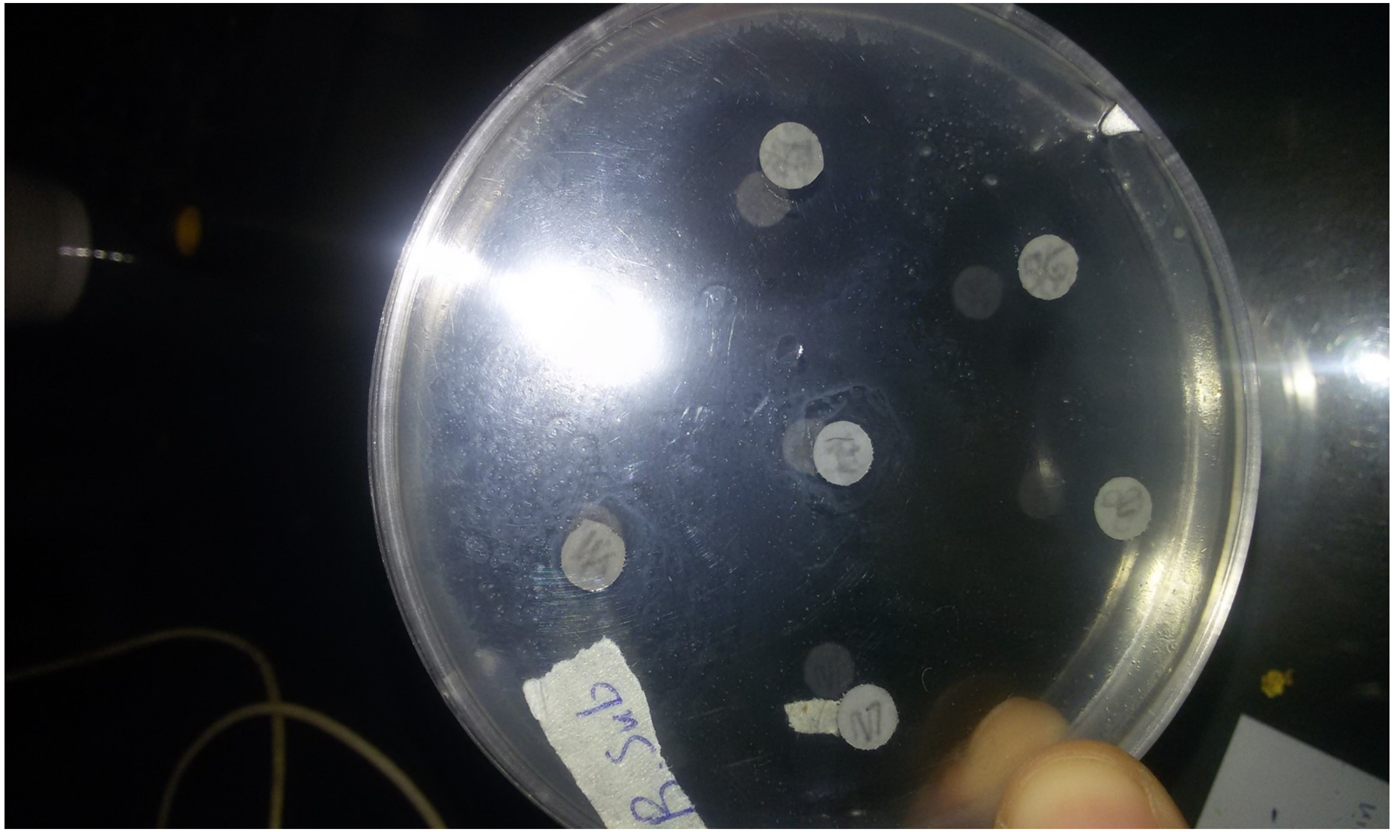

**Figures S22.** Antibacterial activity of compounds against pathogenic bacterial strains *Escherichia coli, Staphylococcus haemolyticus, Kocuria kristinae, Enterococcus casseliflavus,* and *Bacillus cereus*.

Card Type: GP Testing Instrument: [illegible]

Bionumber: 050402123720221
Organism Quantity:

| | |
|---|---|
| **Comments:** | |

| | | | | | | |
|---|---|---|---|---|---|---|
| Identification Information | **Card:** | GP | **Lot Number:** | 242381940 | **Expires:** | May 29, 2017 13:00 EEST |
| | **Completed:** | Mar 16, 2017 14:12 EET | **Status:** | Final | **Analysis Time:** | 7.00 hours |
| Selected Organism | 96% Probability | | **Staphylococcus haemolyticus** | | | |
| | **Bionumber:** 050402123720221 | | | | **Confidence:** | Acceptable identification |

| | |
|---|---|
| **SRF Organism** | |
| **Analysis Organisms and Tests to Separate:** | |
| **Analysis Messages:** | |
| **Contraindicating Typical Biopattern(s)** | |
| Staphylococcus haemolyticus | SAC(57),AlaA(2),dGAL(77),POLYB(1), |

Biochemical Details

| | | | | | | | | | | | | | | | | | |
|---|---|---|---|---|---|---|---|---|---|---|---|---|---|---|---|---|---|
| 2 | AMY | - | 4 | PIPLC | - | 5 | dXYL | - | 8 | ADH1 | + | 9 | BGAL | - | 11 | AGLU | + |
| 13 | APPA | - | 14 | CDEX | - | 15 | AspA | - | 16 | BGAR | - | 17 | AMAN | - | 19 | PHOS | (-) |
| 20 | LeuA | - | 23 | ProA | - | 24 | BGURr | - | 25 | AGAL | - | 26 | PyrA | + | 27 | BGUR | - |
| 28 | AlaA | + | 29 | TyrA | - | 30 | dSOR | - | 31 | URE | - | 32 | POLYB | + | 37 | dGAL | + |
| 38 | dRIB | + | 39 | ILATk | + | 42 | LAC | - | 44 | NAG | + | 45 | dMAL | + | 46 | BAD | - |
| 47 | NOVO | - | 50 | NC6.5 | + | 52 | dMAN | - | 53 | dMNE | - | 54 | MBdG | - | 56 | PUL | - |
| 57 | dRAF | - | 58 | O129R | + | 59 | SAL | - | 60 | SAC | - | 62 | dTRE | + | 63 | ADH2s | |
| 64 | OPTO | + | | | | | | | | | | | | | | | |

**Figure (8): Identification report of bacterial isolate (*Staphylococcus haemolyticus*) using VITEK 2 system.**

Bionumber: 120200601563571

Organism Quantity:

| | |
|---|---|
| Comments: | |

| | | | | |
|---|---|---|---|---|
| Identification Information | Card: GP | Lot Number: 242381940 | Expires: | May 29, 2017 13:00 EEST |
| | Completed: Mar 18, 2017 15:59 EET | Status: Final | Analysis Time: | 3.75 hours |
| Selected Organism | 96% Probability | Enterococcus casseliflavus | | |
| | Bionumber: 120200601563571 | | Confidence: | Excellent identification |
| SRF Organism | | | | |

Analysis Organisms and Tests to Separate:

Analysis Messages:

Contraindicating Typical Biopattern(s)

Enterococcus casseliflavus AMAN(24),

Biochemical Details

| | | | | | | | | | | | | | | | | | |
|---|---|---|---|---|---|---|---|---|---|---|---|---|---|---|---|---|---|
| 2 | AMY | + | 4 | PIPLC | - | 5 | dXYL | - | 8 | ADH1 | - | 9 | BGAL | + | 11 | AGLU | - |
| 13 | APPA | - | 14 | CDEX | - | 15 | AspA | - | 16 | BGAR | - | 17 | AMAN | + | 19 | PHOS | - |
| 20 | LeuA | - | 23 | ProA | - | 24 | BGURr | - | 25 | AGAL | - | 26 | PyrA | - | 27 | BGUR | - |
| 28 | AlaA | - | 29 | TyrA | + | 30 | dSOR | + | 31 | URE | - | 32 | POLYB | - | 37 | dGAL | - |
| 38 | dRIB | + | 39 | ILATk | - | 42 | LAC | - | 44 | NAG | + | 45 | dMAL | - | 46 | BACI | [illegible] |
| 47 | NOVO | - | 50 | NC6.5 | + | 52 | dMAN | + | 53 | dMNE | + | 54 | MBdG | + | 56 | PUL | - |
| 57 | dRAF | + | 58 | O129R | - | 59 | SAL | + | 60 | SAC | + | 62 | dTRE | + | 63 | ADH2s | [illegible] |
| 64 | OPTO | + | | | | | | | | | | | | | | | |

**Figure (6): Identification report of bacterial isolate (*Enterococcus casseliflavus*) using VITEK 2 system.**

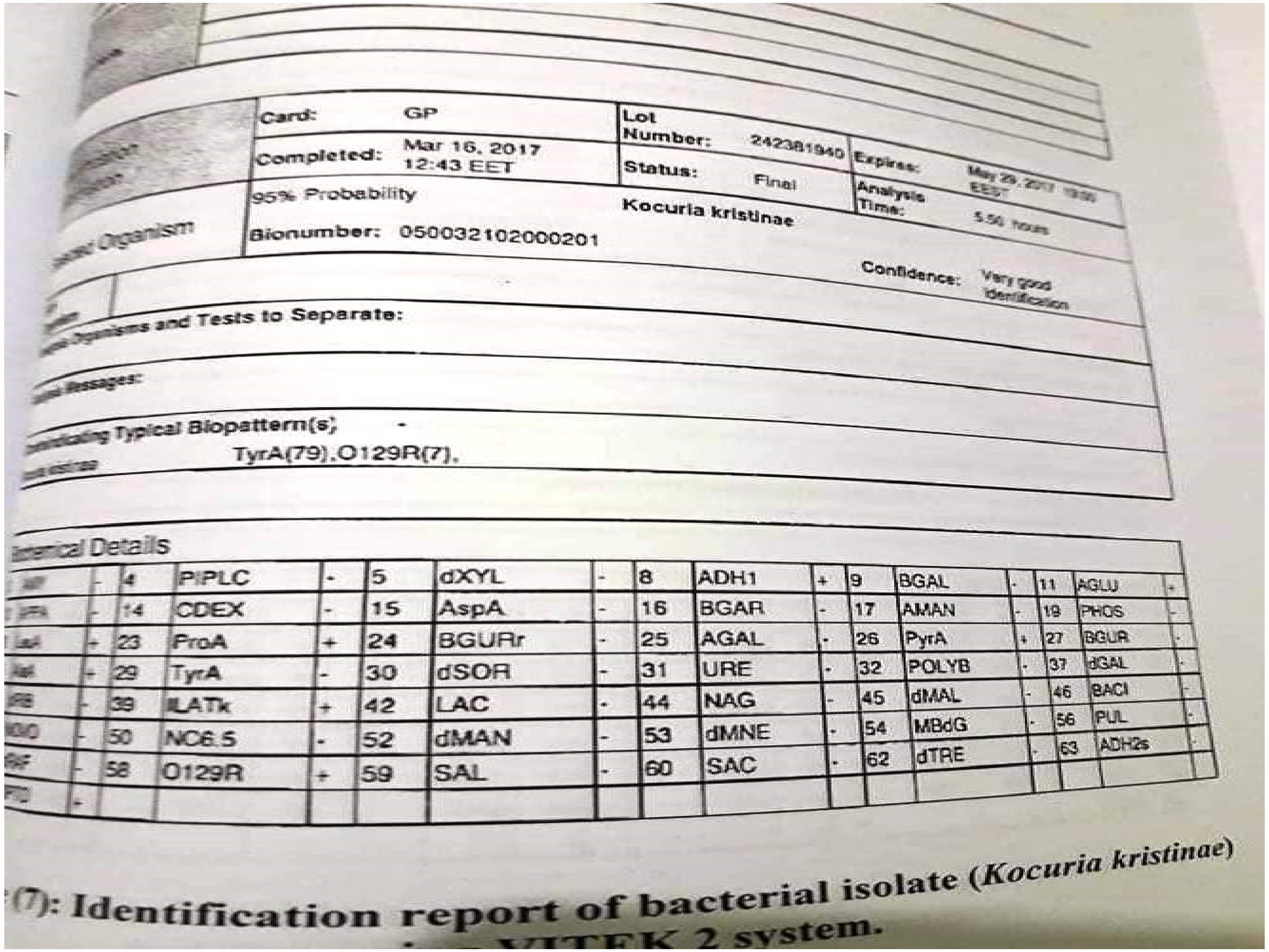

Card: GP

Completed: Mar 16, 2017 12:43 EET

Lot Number: 242381940

Expires: May 29, 2017 [illegible] EEST

Status: Final

Analysis Time: 5.50 hours

[illegible] Organism

95% Probability — Kocuria kristinae

Bionumber: 050032102000201

Confidence: Very good identification

[illegible] Organisms and Tests to Separate:

[illegible] Messages:

[illegible] Typical Biopattern(s)

[illegible] kristinae — TyrA(79),O129R(7),

[illegible] Details

| | | | | | | | | | | | | | | | | | | | |
|---|---|---|---|---|---|---|---|---|---|---|---|---|---|---|---|---|---|---|---|
| [illegible] | - | 4 | PIPLC | - | 5 | dXYL | - | 8 | ADH1 | + | 9 | BGAL | - | 11 | AGLU | [illegible] |
| [illegible] | - | 14 | CDEX | - | 15 | AspA | - | 16 | BGAR | - | 17 | AMAN | - | 19 | PHOS | - |
| [illegible] | + | 23 | ProA | + | 24 | BGURr | - | 25 | AGAL | - | 26 | PyrA | + | 27 | BGUR | - |
| [illegible] | + | 29 | TyrA | - | 30 | dSOR | - | 31 | URE | - | 32 | POLYB | - | 37 | dGAL | - |
| [illegible] | - | 39 | ILATk | + | 42 | LAC | - | 44 | NAG | - | 45 | dMAL | - | 46 | BACI | - |
| [illegible] | - | 50 | NC6.5 | - | 52 | dMAN | - | 53 | dMNE | - | 54 | MBdG | - | 56 | PUL | - |
| [illegible] | - | 58 | O129R | + | 59 | SAL | - | 60 | SAC | - | 62 | dTRE | - | 63 | ADH2s | - |
| [illegible] | - | | | | | | | | | | | | | | | |

[illegible](7): Identification report of bacterial isolate (*Kocuria kristinae*) [illegible] VITEK 2 system.

**Figure S23.** BIOMERIEUX VITEK2 SYSTEM-bio number analysis
